# Supplementary material for: A cis‐acting bidirectional transcription switch controls sexual dimorphism in the liverwort
Source: EMBO J. 2019 Jan 4;38(6):e100240. doi: 10.15252/embj.2018100240 (PMC6418429; doi:10.15252/embj.2018100240)
Supplement: Supplementary file 1 — Appendix [file EMBJ-38-e100240-s001.pdf]

Appendix for

A *cis*-acting bidirectional transcription switch controls sexual dimorphism in the liverwort

Tetsuya Hisanaga, Keitaro Okahashi, Shohei Yamaoka, Tomoaki Kajiwara, Ryuichi Nishihama, Masaki Shimamura, Katsuyuki T. Yamato, John L. Bowman, Takayuki Kohchi and Keiji Nakajima

Table of contents

Figure S1 Generation of loss-of-function Mp*fgmyb* alleles.

Figure S2 Vegetative growth of Mp*fgmyb* mutants is indistinguishable from that of wild-type plants.

Figure S3 Characterization of Mp*FGMYB* and *SUF* transcripts by 5' and 3' RACE PCRs

Figure S4 Generation of loss-of-function *suf* alleles.

Supplementary Methods

References

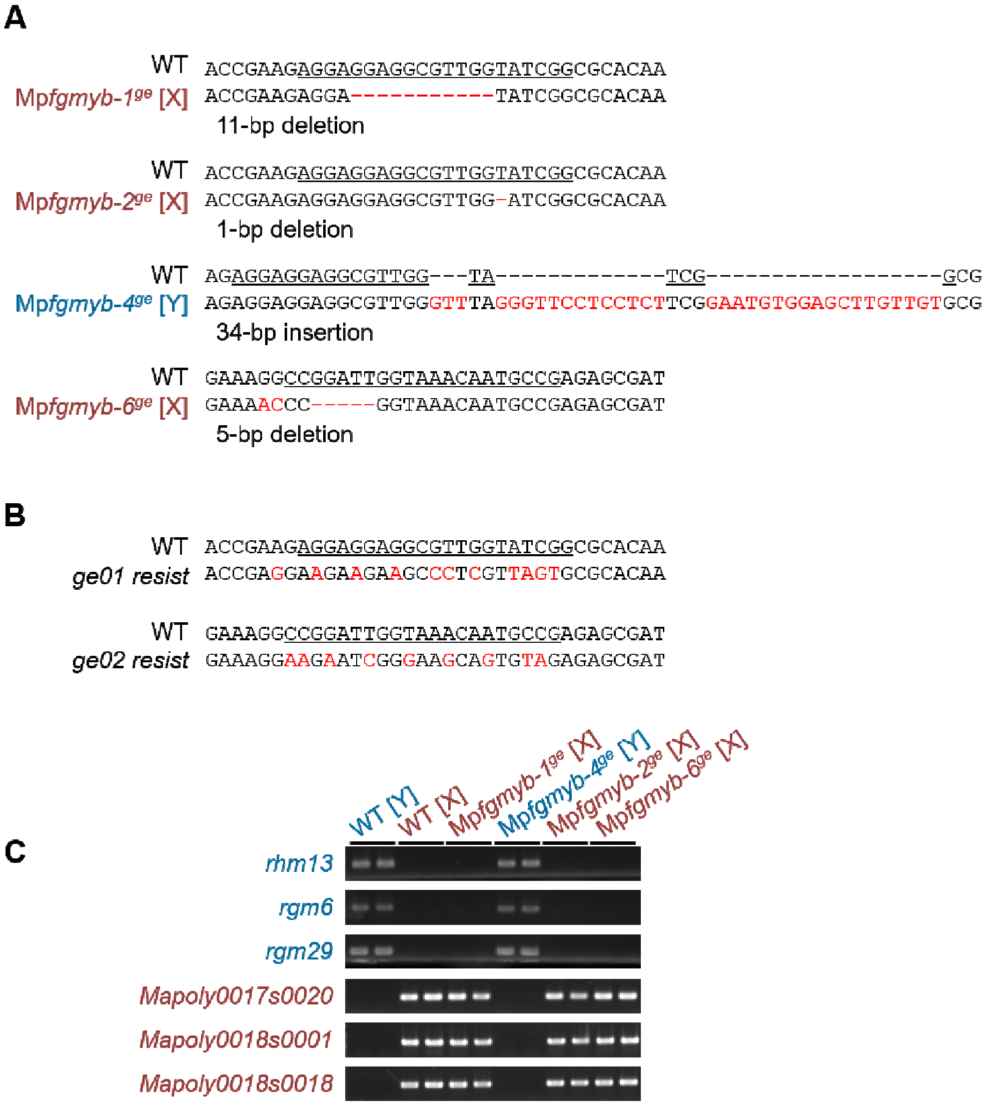

Figure S1 - Generation of loss-of-function Mp*fgmyb* alleles

A Sequence alignments of wild-type and mutant alleles of Mp*FGMYB*.

B Sequence alignments of wild-type and modified Mp*FGMYB* with synonymous mutations to resist gRNA-mediated Cas9 activity. Mismatched nucleotides are marked in red. Underlines indicate the gRNA target sequences.

C Genomic PCR analyses of sex chromosome-linked DNA markers other than those shown in Fig 3B, indicating the intact nature of the sex chromosomes in Mp*fgmyb* mutants.

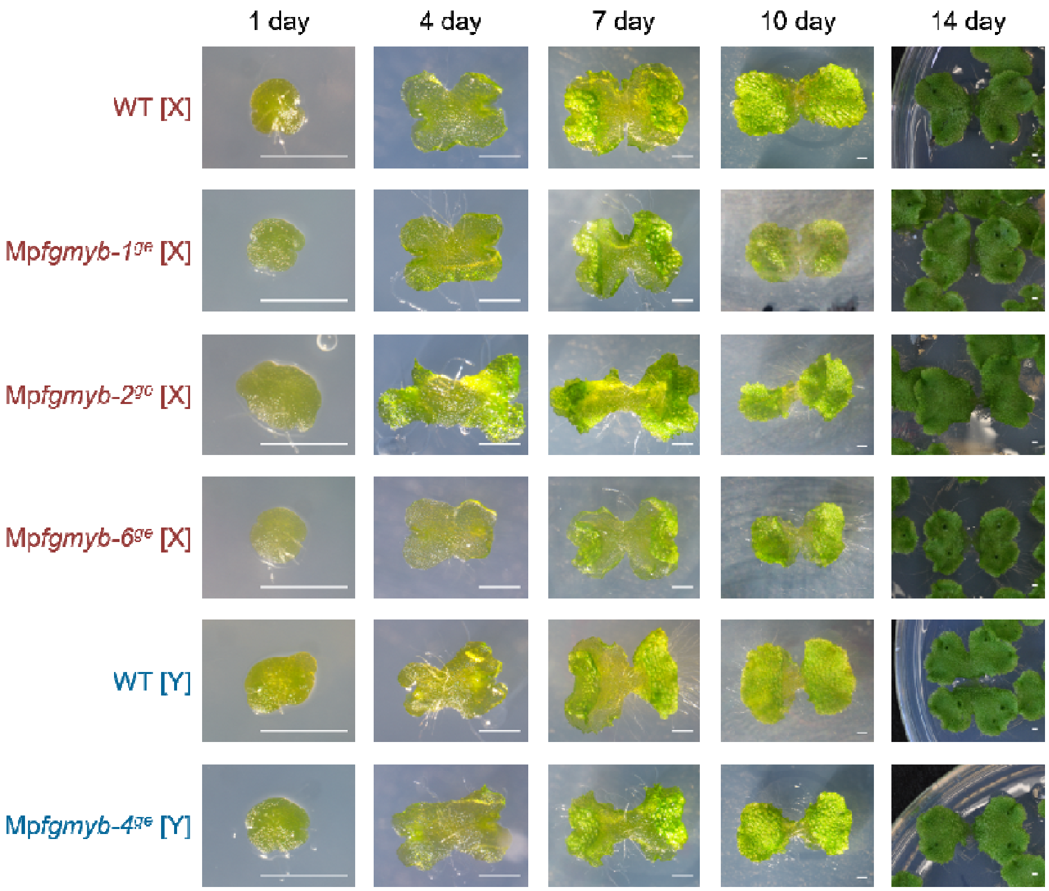

Figure S2 - Vegetative growth of Mp*fgmyb* mutants is indistinguishable from that of wild-type plants

Gross morphologies from the gemma to the vegetative thallus are presented for the indicated time points. Growth was recorded sequentially for the same individuals from day 1 to day 10. Scale bars, 1 mm.

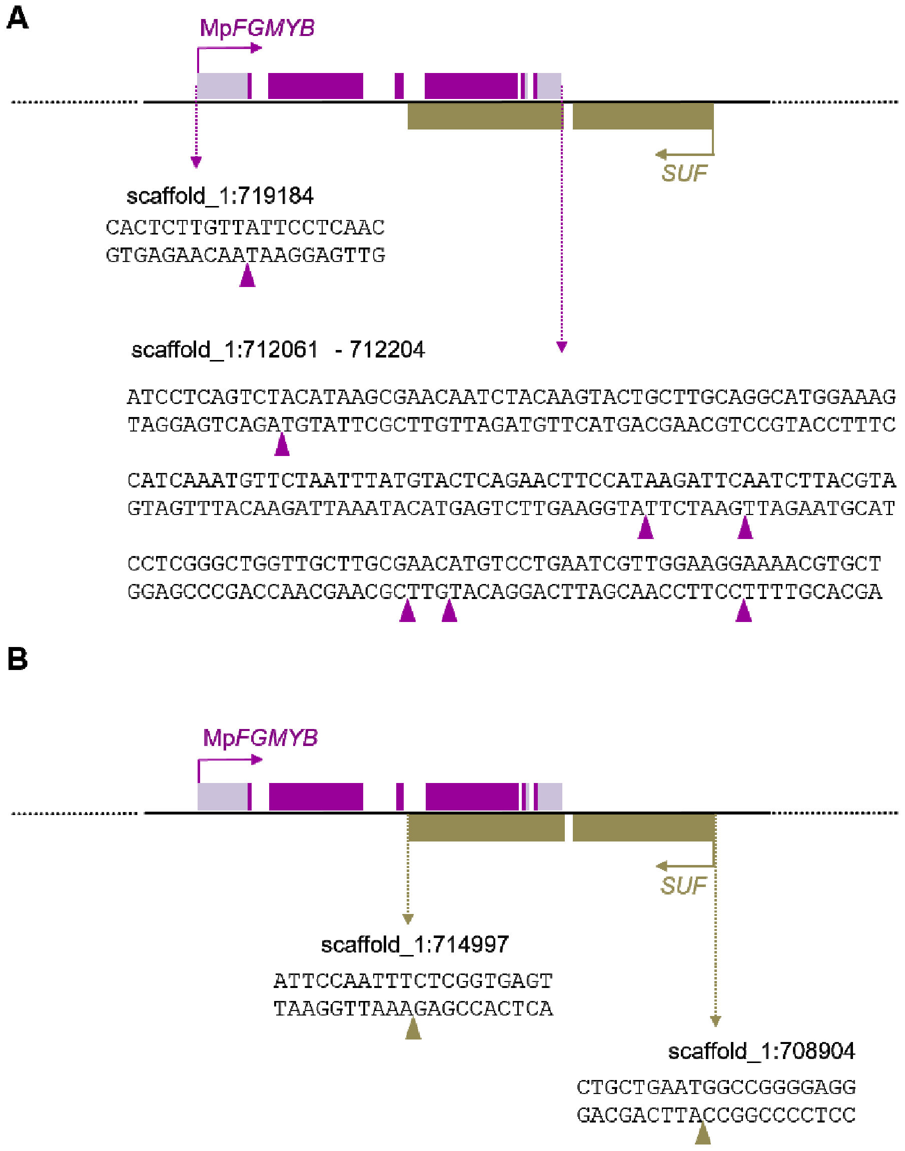

Figure S3 - Characterization of Mp*FGMYB* and *SUF* transcripts by 5' and 3' RACE PCRs

Positions of a transcription start site and polyadenylation sites of Mp*FGMYB* (A) and *SUF* (B) transcripts are plotted on the genome sequences (arrowheads). For Mp*FGMYB*, three and seven clones were sequenced for 5' and 3' RACE products, respectively. For *SUF*, RACE PCR products were directly sequenced based on their apparent homogeneity and the sequencing results indeed indicated invariable 5' and 3' ends of the cDNA. See Supplementary Methods below for details.

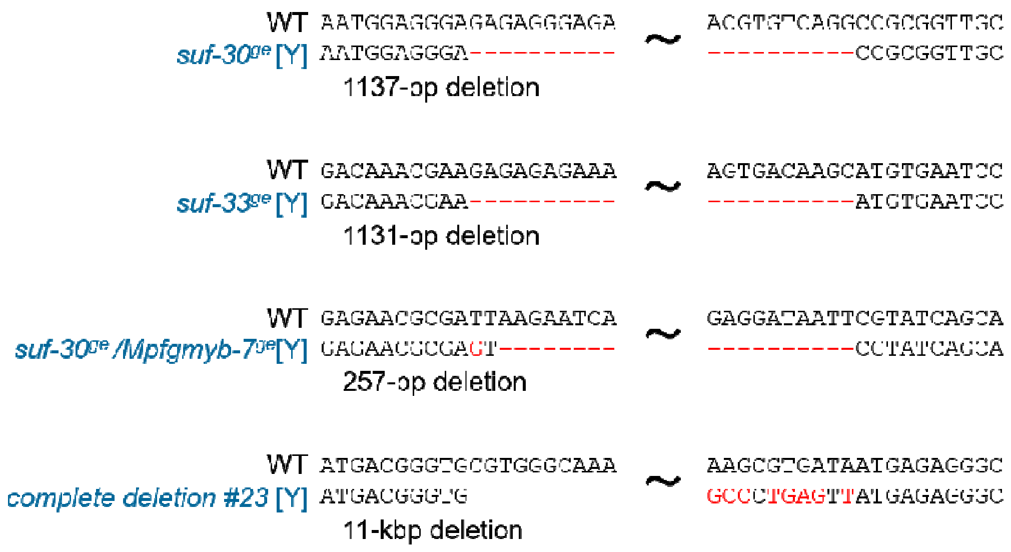

Figure S4 – Generation of loss-of-function *suf* alleles

Sequence alignments of wild-type and mutant *suf* alleles. Deleted and mismatched nucleotides are shown in red.

Supplementary Methods

Quantitative real-time RT-PCR in Fig 2D, 6B and Fig EV4A

Total RNA was extracted from vegetative thalli, gametangiophores and sporophytes using TRIzol reagent (Thermo Ficher Scientific, Waltham, MA) according to the manufacturer's instruction. First-strand cDNA was synthesized using ReverTra Ace (Toyobo, Osaka, Japan) with a 20-mer oligo dT primer. Quantitative real-time RT-PCR were carried out with a CFX96 real-time PCR detection system (Bio-Rad Laboratories, Hercules, CA), using the primer sets listed in Table EV2, and under the following cycle conditions: initial incubation at 95°C for 2 min, followed by 40 cycles of 95°C for 10 s, and 60°C for 30 s.

RT-PCR in Fig 4D and EV3A

Total RNA was extracted from gametangiophores using the RNeasy Plant Mini Kit (Qiagen, Hilden, Germany) with RNase-Free DNase Set (Qiagen). First-strand cDNA was synthesized using the Prime Script RT Reagent Kit with gDNA Eraser (TaKaRa Bio, Shiga, Japan). A mixture of random primers and oligo-dT primers included in the kit was used for cDNA synthesis. PCR analysis was carried out using Quick Taq polymerase (TOYOBO) and the primer sets listed in Table EV2, under the following cycle conditions: initial incubation at 98°C for 2 min, followed by 30 cycles of 94°C for 30 s, 55°C for 30 s, and then 68°C for 40 s.

RT-PCR in Fig 5B

M. polymorpha plants were grown under continuous white light (cW) for 10 days, or under cW for 10 days followed by three days of cW and far-red light (about 40~50 μmol m^{-2} s^{-1}). RNA was extracted from gametangiophores and apical notch regions of thalli as described previously (Inoue et al, 2016) and amplified using KOD FX Neo polymerase (TOYOBO). Mp*FGMYB* was amplified with the primer pair MpFGMYB Seq7F/MpFGMYB qPCR R, and under the following PCR conditions: initial incubation at 98°C for 2 min, followed by 38 cycles of 96°C for 10 s, 60°C for 20 s, and 68°C for 1 min. *SUF* transcripts were amplified using the primer pair SUFSeqF5 and SUFSeqR5, under the following PCR conditions: initial incubation at 98°C for 2 min, followed by

37 cycles of 96°C for 10 s, 60°C for 20 s, and 68°C for 2 min. As a control, Mp*EF1α* transcripts were amplified using the primer pair Mplg01fw/Mplg01rv, using the following conditions: initial incubation at 98°C for 2 min, followed by 22 cycles of 96°C for 10 s, 58°C for 20 s, and 68°C for 1 min.

Quantitative real-time RT-PCR in Fig 6D

Total RNA was extracted from gametangiophores using the RNeasy Plant Mini Kit with RNase-Free DNase Set. First-strand cDNA was synthesized using the Prime Script RT Reagent Kit with gDNA Eraser. Gene-specific primers carrying adaptor sequences, listed in Table EV2 were used for cDNA synthesis. Quantitative real-time RT-PCR was carried out with the SYBR Premix Ex Taq II (TaKaRa Bio) and the primer sets listed in Table EV2 using the LightCycler96 (Roche Diagnostics, Basel, Switzerland), under the following cycle conditions: initial incubation at 95°C for 1 min, followed by 45 cycles of 95°C for 10 s, 60°C for 10 s and then 72°C for 10 s.

RT-PCR in Fig EV4B

Total RNA was extracted from gametangiophores and apical notch regions of thalli using the RNeasy Plant Mini Kit with RNase-Free DNase Set. First-strand cDNA was synthesized using the Prime Script RT Reagent Kit with gDNA Eraser. Gene-specific primers carrying adaptor sequences listed in Table EV2 were used for cDNA synthesis. PCR analysis was carried out using Quick Taq polymerase and the primer sets listed in Table EV2, under the following cycle conditions: initial incubation at 98°C for 2 min, followed by 30 cycles of 94°C for 30 s, 55°C for 30 s, and then 68°C for 40 s.

RT-PCR in Fig EV5

Total RNA was extracted from gametangiophores as described previously (Inoue et al, 2016) and amplified using KOD FX Neo polymerase. Sex-specific transcripts were detected using the primer sets listed in Table EV2 under the following PCR conditions: initial incubation at 98°C for 20 s, followed by 30 cycles, or 35 cycles for Mp*LC7*, of 98°C for 30 s, 58°C for 30 s, and 68°C for 40 s, and additional incubation at 68°C for 2 min.

5’ RACE PCR

Total RNAs extracted from archegoniophores and antheridiophores were reverse-transcribed using ReverTra Ace with the primer MpFGMYB SP1 or SUF SP1. cDNAs thus obtained were polyadenylated by terminal transferase (New England Biolabs Japan, Tokyo, Japan) and then used as templates for consecutive PCRs. First PCR was carried out using KOD FX neo polymerase and the primer pairs of Oligo dT-Anchor /MpFGMYB SP2 for *MpFGMYB* or Oligo dT-Anchor/SUF SP2 for *SUF* under the following cycle conditions: 94°C for 2 min, followed by 35 cycles of 98°C for 10 s, 65°C (Mp*FGMYB*) or 63°C (*SUF*) for 15 s, and then 68°C for 40 s. Second PCR for *SUF* was carried out using KOD FX neo, diluted first PCR products and the primer pair PCR Anchor/SUF SP3 under the following cycle conditions: 94°C for 2 min, followed by 35 cycles of 98°C for 10 s, 61°C for 15 s, and then 68°C for 40 s. The second PCR products were purified by gel extraction and directly sequenced using the SUF SP3 primer. Second PCR for Mp*FGMYB* was carried out using Ex Taq (TaKaRa Bio Co.), diluted first PCR products and the primer pair PCR Anchor /MpFGMYB SP3 under the following cycle conditions: 94°C for 2 min, followed by 35 cycles of 98°C for 10 s, 65°C for 15 s, and then 72°C for 20 s. The second PCR products were purified by gel extraction and cloned into pGEM-T Easy vector (Promega, Madison, WI), before sequencing.

3’ RACE PCR

To isolate 3’ regions of Mp*FGMYB* and *SUF* transcripts, total RNAs were extracted from archegoniophores and antheridiophores, respectively, and used for cDNA synthesis using the OligodT-adaptor primer. Mp*FGMYB* fragment was amplified using Quick Taq and the primer pair 3RACE-adaptor/MpMYB119seq13 under the following cycle conditions: 94°C for 2 min, followed by 30 cycles of 94°C for 30 s, 55°C for 30 s, and then 68°C for 60 s. PCR products were purified by ethanol precipitation and cloned into the pGEM-T Easy vector, before sequencing with the M13Fw primer. *SUF* fragments were amplified using the KOD one polymerase (TOYOBO) and the primer pair 3RACE-adaptor/SUF SP4 under the following cycle conditions: 94°C for 2 min, followed by 30 cycles of 98°C for 10 s, 57°C for 5 s, and then 68°C for 1 s. Second PCR was carried out using the primer pair 3RACE-adaptor/SUF SP5 under the same

cycle condition as the for the first PCR. The third PCR was carried out using the Quick Taq and the primer pair 3RACE-adaptor/SUF SP6 under the following cycle conditions: 94°C for 2 min, followed by 30 cycles of 94°C for 30 s, 58°C for 30 s, and then 68°C for 60 s. PCR products were purified by gel extraction and cloned into pGEM-T Easy vector before sequencing.

DNA construction

pMpGE010_MpFGMYBge (for CRISPR/Cas9-mediated genome editing)

To disrupt MpFGMYB using CRISPR/Cas9-mediated targeted genome editing, three target sites in the MYB domain-coding region were selected for the design of gRNAs. DNA fragments used to generate the three gRNA species were prepared by annealing three pairs of synthetic oligo nucleotides (MpFGMYBge01Fw/MpFGMYBge01Rv, MpFGMYBge02Fw/MpFGMYBge02Rv, and MpFGMYBgRNA4F/ MpFGMYBgRNA4R). The fragments were then inserted into the BsaI site of pMpGE_En03 (Cat.N. 71535, Addgene, Cambridge, MA) to yield pMpGE_En03-MpFGMYBge01, pMpGE_En03-MpFGMYBge02, and pMpGE_En03-MpFGMYBgRNA4, respectively, and then transferred into pMpGE010 (Cat.N. 71536, Addgene) (Sugano et al, 2018) using the Gateway LR reaction (Thermo Fisher Scientific) to generate pMpGE010_MpFGMYBge01, pMpGE010_MpFGMYBge02, and pMpGE010_MpFGMYBgRNA4, respectively.

MpFGMYBpro:Citrine-NLS:3'MpFGMYB (for expression analyses)

A 4-kb genomic fragment spanning the 3-kb of 5' upstream sequence and a 1-kb 5′-UTR of Mp*FGMYB* was amplified from the Tak-2 genomic DNA using the primers H-MpFGMYBpro-Fw and PmeI-MpFGMYBpro-Rv. A 5.7-kb fragment containing the 0.7-kb 3′-UTR and a 5-kb 3′-flanking sequence of Mp*FGMYB* was amplified from Tak-2 genomic DNA using the primers PmeI-MpFGMYBter-Fw and MpFGMYB3′-Rv. The two fragments were assembled into the SmaI site of the pAN19 vector (a modified pUC19 vector kindly provided by Dr. Takehide Kato) using a SLiCE reaction (Motohashi, 2015; Zhang et al, 2012) to yield pAN19-MpFGMYBpro-PmeI-3′MpFGMYB. A Citrine-NLS-coding sequence was amplified from the pMpGWB115 (Ishizaki et al, 2015) vector using the primers

FGMYBp-Cit-Fw and FGMYBt-NLS-Rv. This fragment was assembled into the PmeI site of pAN19-MpFGMYBpro-PmeI-3′MpFGMYB in a SLiCE reaction, yielding pAN19-MpFGMYBpro-Citrine-NLS-3′MpFGMYB. A *lacZ* gene with multi-cloning sites was amplified from the pBIN31 vector with the primers PmeI-lacz-Fw and AscI-lacz-Rv. A Gateway cassette of pMpGWB301 (Ishizaki et al, 2015) was removed by digestion with PmeI and AscI, and then replaced with the *lacZ* fragment in a SLiCE reaction to generate pMpSL30. The MpFGMYBpro-Citrine-NLS-3′MpFGMYB fragments were excised from pAN19-MpFGMYBpro-Citrine-NLS-3′MpFGMYB by digestion with AscI and inserted into the pMpSL30 to yield pMpSL30-MpFGMYBpro-Citrine-NLS-3′MpFGMYB.

gMpFGMYBresist-NosT and gMpFGMYBresist-Citrine-NosT (for complementation)

A 5-kb genomic fragment spanning the promoter and the second exon of Mp*FGMYB* was amplified from the wild-type Tak-2 genomic DNA using the primers attB1-MpFGMYBpro-Fw and attB2-MpFGMYBpro-Rv, and then cloned into the pDONR19 vector (kindly provided by Dr. Satoshi Fujita) using the Gateway BP reaction (Thermo Fisher Scientific) to yield pDONR19-MpFGMYBpro. A 4.5-kb fragment spanning the entire exon and intron regions of Mp*FGMYB* was amplified as two separate fragments from Tak-2 genomic DNA using the primer pairs MpFGMYB-N1-Fw/MpFGMYB-N1-Rv and MpFGMYB-C1-Fw/MpFGMYB-C1-Rv. The pDNOR19-MpFGMYBpro vector was linearized by PCR with the primers MpFGMYBpro-Rv and attB2-Fw. These three fragments were assembled in a SLiCE reaction to yield pDONR19-gMpFGMYBresist01, which contained synonymous mutations to resist being targeted by the ge01 gRNA. An alternative version (pDONR19-gMpFGMYBresist02), resistant to the ge02 gRNA, was constructed similarly with the primers MpFGMYB-N1-Fw, MpFGMYB-N2-Rv, MpFGMYB-C2-Fw, and MpFGMYB-C1-Rv. The two fragments were transferred into pMpGWB301 and pMpGWB307 (Ishizaki et al, 2015) in a Gateway LR reaction to yield gMpFGMYBresist01-NosT, gMpFGMYBresist02-NosT, gMpFGMYBresist01-Citrine-NosT, and gMpFGMYBresist02-Citrine-NosT.

gMpFGMYBresist-3′MpFGMYB-L and gMpFGMYBresist-Citrine-3′MpFGMYB-L (for complementation)

A 5.4-kb fragment spanning the entire exon and intron region of Mp*FGMYB* was amplified from gMpFGMYBresist01-NosT using the primers gMpFGMYB-Fw and gMpFGMYB-Rv. This fragment was inserted into the PmeI site of pAN19-MpFGMYBpro-PmeI-3′MpFGMYB in a SLiCE reaction to yield pAN19-MpFGMYBpro-gMpFGMYBresist01-3′MpFGMYB. pAN19-MpFGMYBpro-gMpFGMYBresist02-3′MpFGMYB was constructed in the same way, using gMpFGMYBresist02-NosT as a template. A 6.1-kb fragment containing the entire exon and intron region of Mp*FGMYB* and the Citrine-coding sequence was amplified from gMpFGMYBresist01-Citrine-NosT using the primers gMpFGMYB-Fw and Cit-Rv. This PCR product was inserted into the PmeI site of pAN19-MpFGMYBpro-PmeI-3′MpFGMYB in a SLiCE reaction to yield pAN19-MpFGMYBpro-gMpFGMYBresist01-Citrine-3′MpFGMYB. pAN19-MpFGMYBpro-gMpFGMYBresist02-Citrine-3′MpFGMYB was constructed in the same way using gMpFGMYBresist02-Citrine-NosT as a template. These four fragments were excised as an AscI fragment and inserted into pMpSL30.

gMpFGMYB-S

A 4-kb genomic fragment spanning the 3-kb 5’ upstream sequence and a 1-kb 5′-UTR of Mp*FGMYB* was amplified from the Tak-2 genomic DNA using the primers H-MpFGMYBpro-Fw and PmeI-MpFGMYBpro-Rv. A 3.7-kb fragment containing the 0.7-kb 3′-UTR and a 3-kb 3′-flanking sequence of Mp*FGMYB* was amplified from Tak-2 genomic DNA using the primers PmeI-MpFGMYBter-Fw and MpFGMYB3’-S-Rv. The two fragments were assembled at the SmaI site of the pAN19 vector using a SLiCE reaction to yield pAN19-MpFGMYBpro-PmeI-3′MpFGMYB-S. A 5.4-kb fragment spanning the entire exon and intron region of Mp*FGMYB* was amplified from gMpFGMYBresist01-NosT using the primers gMpFGMYB-Fw and gMpFGMYB-Rv. This fragment was inserted into the PmeI site of pAN19-MpFGMYBpro-PmeI-3′MpFGMYB-S in a SLiCE reaction to yield pAN19-MpFGMYBpro-gMpFGMYBresist01-3′MpFGMYB-S. This fragment was

excised as an AscI fragment and inserted into pMpSL30 to yield pMpSL30-gMpFGMYB-S.

Plasmids for the disruption of the SUF transcription start site

To construct a plasmid that disrupts the *SUF* transcription start site using genome editing, the oligonucleotide pair SUFgRNA1F and SUFgRNA1R was annealed and subcloned into the BsaI site of pMpGE_En03 to yield pMpGE_En03-SUFgRNA1. In parallel, a fragment containing a BsaI site and the Mp*U6* promoter was excised from pMpGE_En03 by digestion with EcoRI and NotI, and then subcloned into pBCSK^{+}. An EcoRI site was added to the resulting plasmid by digestion with SacI and SacII, followed by insertion of an adaptor DNA segment containing an EcoRI site. The oligonucleotide pair SUFgRNA2F and SUFgRNA2R was annealed and subcloned into the Bsa I site of this plasmid. The resulting Mp*U6* promoter-SUFgRNA2 fragment was excised by digestion with EcoRI and inserted into the EcoRI site of pMpGE_En03-SUFgRNA1. The resulting DNA fragment containing two Mp*U6* promoter-gRNA cassettes was transferred into pMpGE018 using the Gateway LR reaction, and used for genome editing with the CRISPR/Cas9^{D10A} system. pMpGE018 and the CRISPR/Cas9^{D10A} system were developed with Dr. Keisuke Inoue.

Plasmids for the complete deletion of the MpFGMYB/SUF locus

To delete the entire Mp*FGMYB*/*SUF* gene locus, pairs of oligonucleotides were designed along the promoter region of Mp*FGMYB* and *SUF*. After annealing, these four gRNA sequences (MpFGMYBgRNA1, MpFGMYBgRNA2, SUFgRNA3, and SUFgRNA4) were subcloned into the BsaI sites of pMpGE_En04, pBC-GE12, pBC-GE23, and pBC-GE34, respectively, and further subcloned into pMpGE_En04. The resulting gRNA sequences were transferred into pMpGE018 using the Gateway LR reaction.

Plasmid for SUF overexpression

A genomic fragment corresponding to the *SUF* transcription unit was amplified from Tak-1 genomic DNA using the primer pair of CUFF.93_OX_F1/CUFF.93_OX_R2, and cloned into the pMpGWB103 vector (Ishizaki et al, 2015) using Gateway technology.

gAtMYB64-Citrine

A 1.8-kb genomic fragment spanning the regulatory sequences of At*MYB64* was amplified from the Col-0 genomic DNA using the primers H-AtMYB64pro-Fw and AtMYB64pro-Rv. A 0.7-kb fragment of the 3' flanking sequence from At*MYB64* was amplified from Col-0 genomic DNA using the primers M-AtMYB64ter-Fw and E-AtMYB64ter-Rv. The two fragments were assembled into the SmaI site of the pAN19 vector using a SLiCE reaction (Motohashi, 2015; Zhang et al, 2012) to yield pAN19-AtMYB64pro-SmaI-AtMYB64ter. A Citrine-coding sequence was amplified from the pMpGWB108 (Ishizaki et al, 2015) vector using the primers M64-Sma-linker-Fw and M64-Nru-Cit-Rv. This fragment was assembled into the SmaI site of pAN19-AtMYB64pro-SmaI-AtMYB64ter in a SLiCE reaction, yielding pAN19-AtMYB64pro-SmaI-Cit-NruI-AtMYB64ter. A 1.5-kb fragment spanning the entire exon and intron region of At*MYB64* was amplified from Col-0 genomic DNA using the primers M64-AtMYB64-Fw and Linker-AtMYB64-Rv. This fragment was inserted into the SmaI site of pAN19-AtMYB64pro-SmaI-Cit-NruI-AtMYB64ter in a SLiCE reaction to yield pAN19-gAtMYB64-Citrine. The gAtMYB64-Citrine fragment was excised from pAN19- gAtMYB64-Citrine by digestion with AscI and inserted into the pBIN41 to yield pBIN41- gAtMYB64-Citrine.

References

Inoue K, Nishihama R, Kataoka H, Hosaka M, Manabe R, Nomoto M, Tada Y, Ishizaki K, Kohchi T (2016) Phytochrome signaling Is mediated by PHYTOCHROME INTERACTING FACTOR in the liverwort *Marchantia polymorpha*. *Plant Cell* **28:** 1406-1421

Ishizaki K, Nishihama R, Ueda M, Inoue K, Ishida S, Nishimura Y, Shikanai T, Kohchi T (2015) Development of Gateway binary vector series with four different selection markers for the liverwort *Marchantia polymorpha*. *PLoS One* **10:** e0138876

Motohashi K (2015) A simple and efficient seamless DNA cloning method using SLiCE from *Escherichia coli* laboratory strains and its application to SLiP site-directed mutagenesis. *BMC Biotechnol* **15:** 47

Sugano SS, Nishihama R, Shirakawa M, Takagi J, Matsuda Y, Ishida S, Shimada T, Hara-Nishimura I, Osakabe K, Kohchi T (2018) Efficient CRISPR/Cas9-based genome editing and its application to conditional genetic analysis in *Marchantia polymorpha*. *PLoS One* **13:** e0205117

Zhang Y, Werling U, Edelmann W (2012) SLiCE: a novel bacterial cell extract-based DNA cloning method. *Nucleic Acids Res* **40:** e55
