# Supplementary material for: A cis‐acting bidirectional transcription switch controls sexual dimorphism in the liverwort
Source: EMBO J. 2019 Jan 4;38(6):e100240. doi: 10.15252/embj.2018100240 (PMC6418429; doi:10.15252/embj.2018100240)
Supplement: Supplementary file 2 — Expanded View Figures PDF [file EMBJ-38-e100240-s002.pdf]

Expanded View Figures

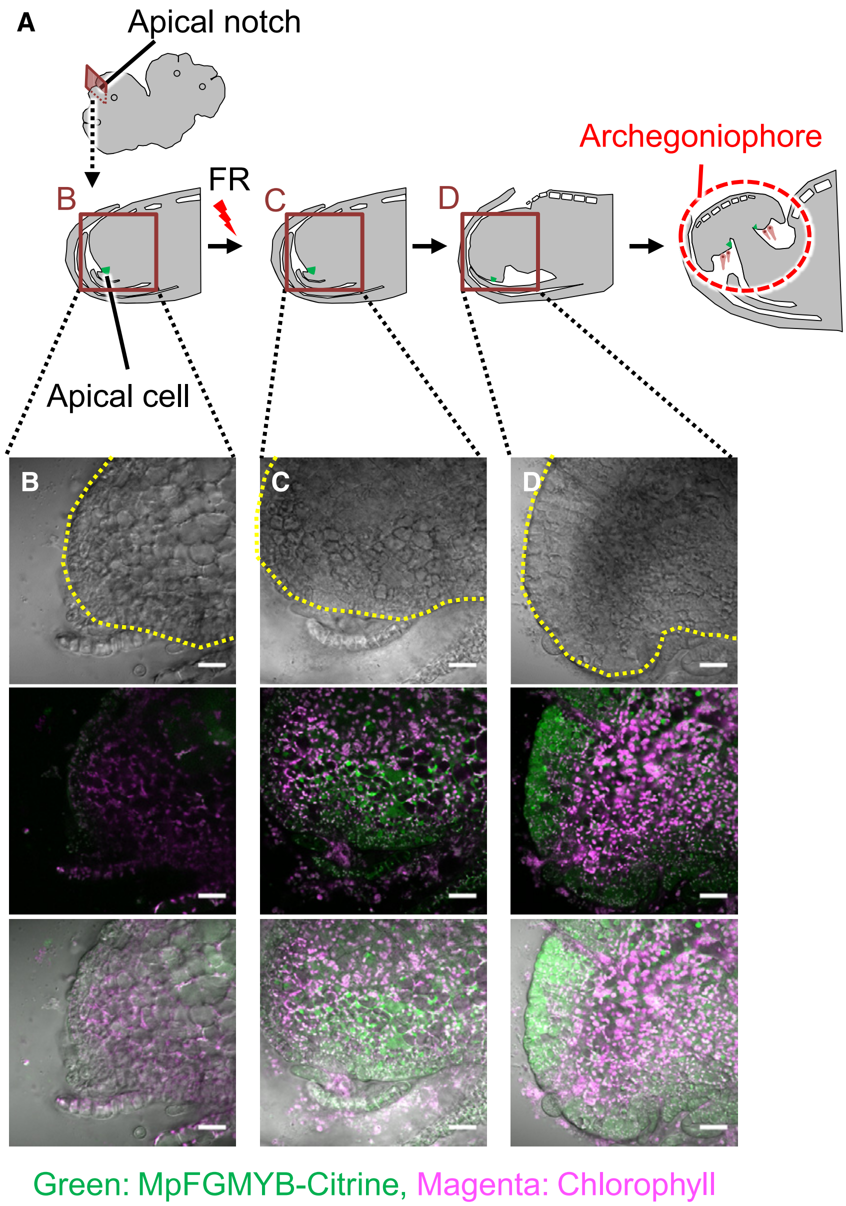

Figure EV1. MpFGMYB expression precedes archegoniophore morphogenesis.

A A schematic representation of the process of archegoniophore morphogenesis after far-red irradiation. Longitudinal sections of apical notch regions are presented. Around 14 days after induction, dome-shaped archegoniophore primordia developed at the ventral side of apical notch area. Regions corresponding to the images shown in (B–D) are boxed.

B–D Confocal microscopic images of the apical notch region of *gMpFGMYBresist-Citrine* plants without (B) or with far-red irradiation (C, D). MpFGMYB-Citrine does not accumulate in the apical notch region of vegetative thalli (B). After 10 days of FR irradiation, MpFGMYB-Citrine accumulates in the ventral side of the apical notch region (C). Expression domain of MpFGMYB-Citrine expands when the morphology of archegoniophore primordia becomes evident (D). Yellow dotted lines delineate the edges of thalli and a developing archegoniophore. Top, bright field images; middle, fluorescent images; bottom, merged images. Scale bar, 25 μm.

© 2018 The Authors

© 2018 The Authors

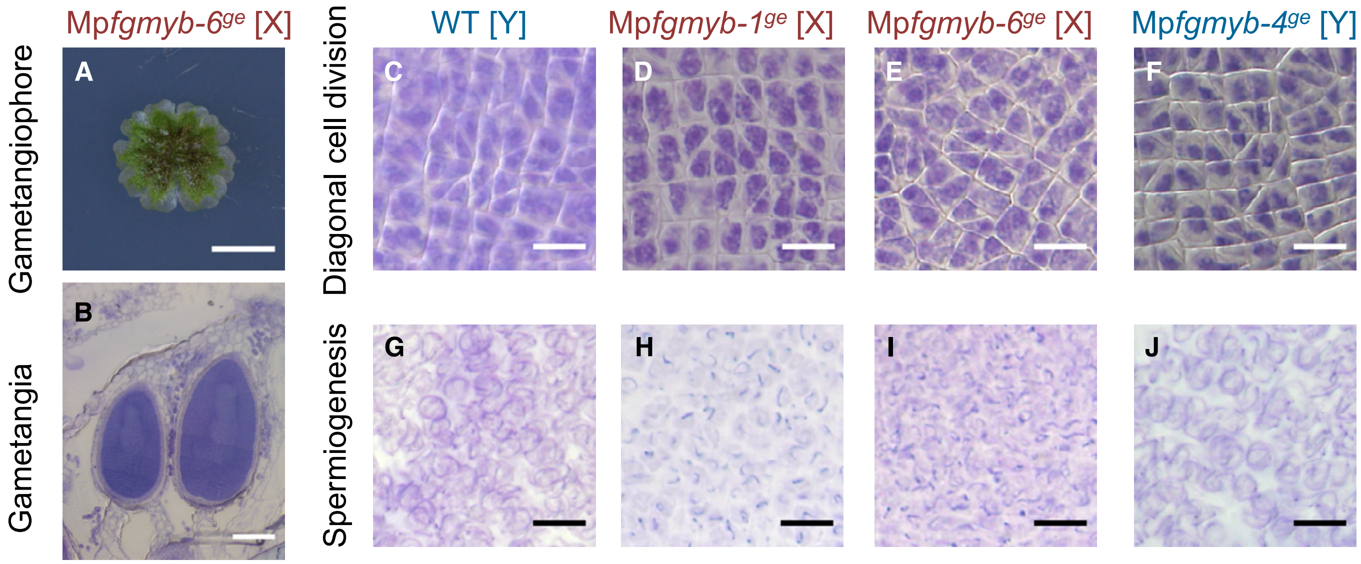

Figure EV2. Multiple Mp*fgmyb* [X] alleles consistently exhibit the female-to-male sex conversion phenotype.

A Gross morphology of antheridiophores developed in Mp*fgmyb-6*^{ge} [X].

B–J Histological analyses indicating antheridium formation (B), diagonal cell division of spermatogenous cells (C–F), and subsequent spermiogenesis (G–J) in the wild-type (C, G), two independent Mp*fgmyb* [X] mutants (D, E, H, and I), and one Mp*fgmyb* [Y] mutant (F, J).

Data information: Scale bars, 5 mm (A), 100 µm (B), 10 µm (C–J).

◀ **Figure EV3. Characterization of the sex conversion phenotypes of Mp*fgmyb* [X].**

A RT–PCR analysis demonstrating loss of expression of female-specific autosomal genes in the antheridiophores of Mp*fgmyb* [X]. Note that X chromosome-linked genes are still expressed in Mp*fgmyb* [X] antheridiophores as in the wild-type females, despite their male-like sexual morphologies. Biological duplicates were analyzed for each genotype.

B–D Hoechst-stained wild-type archegonia treated with sperm from wild-type (B), Mp*fgmyb* [X] (C), and Mp*fgmyb* [Y] (D) plants, indicating the inability of Mp*fgmyb* [X] sperm to enter wild-type archegonia. Arrowheads, sperm in archegonial cavity. Dotted lines, egg cells. Scale bars, 10 µm (B′–D′, B″–D″), 50 µm (B–D).

Source data are available online for this figure.

© 2018 The Authors

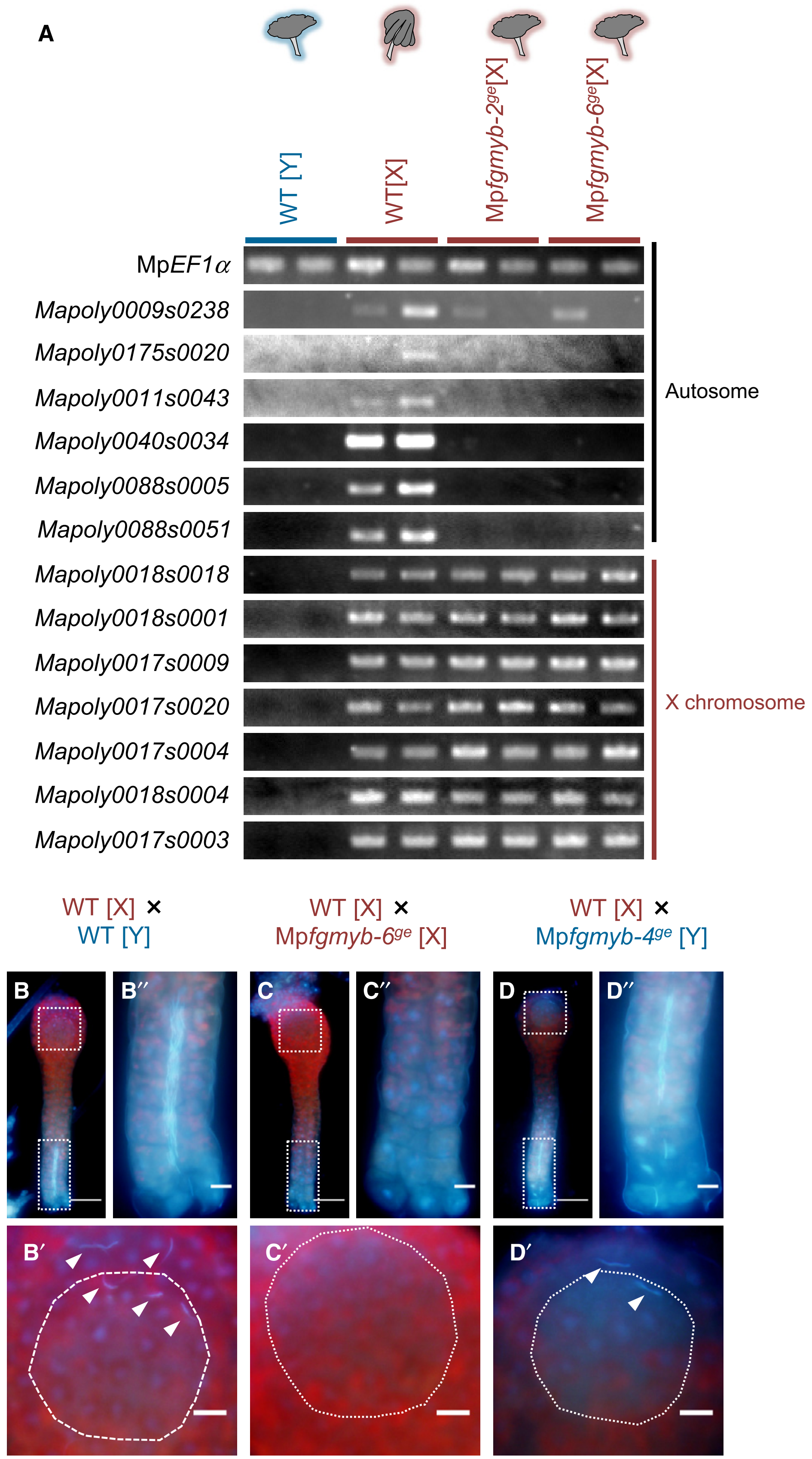
A
WT [Y]
WT[X]
Mpfgmyb-2ge[X]
Mpfgmyb-6ge[X]
MpEF1α
Mapoly0009s0238
Mapoly0175s0020
Mapoly0011s0043
Mapoly0040s0034
Mapoly0088s0005
Mapoly0088s0051
Mapoly0018s0018
Mapoly0018s0001
Mapoly0017s0009
Mapoly0017s0020
Mapoly0017s0004
Mapoly0018s0004
Mapoly0017s0003
Autosome
X chromosome
WT [X] ×
WT [Y]
WT [X] ×
Mpfgmyb-6ge [X]
WT [X] ×
Mpfgmyb-4ge [Y]
B
B″
C
C″
D
D″
B′
C′
D′

Figure EV3.

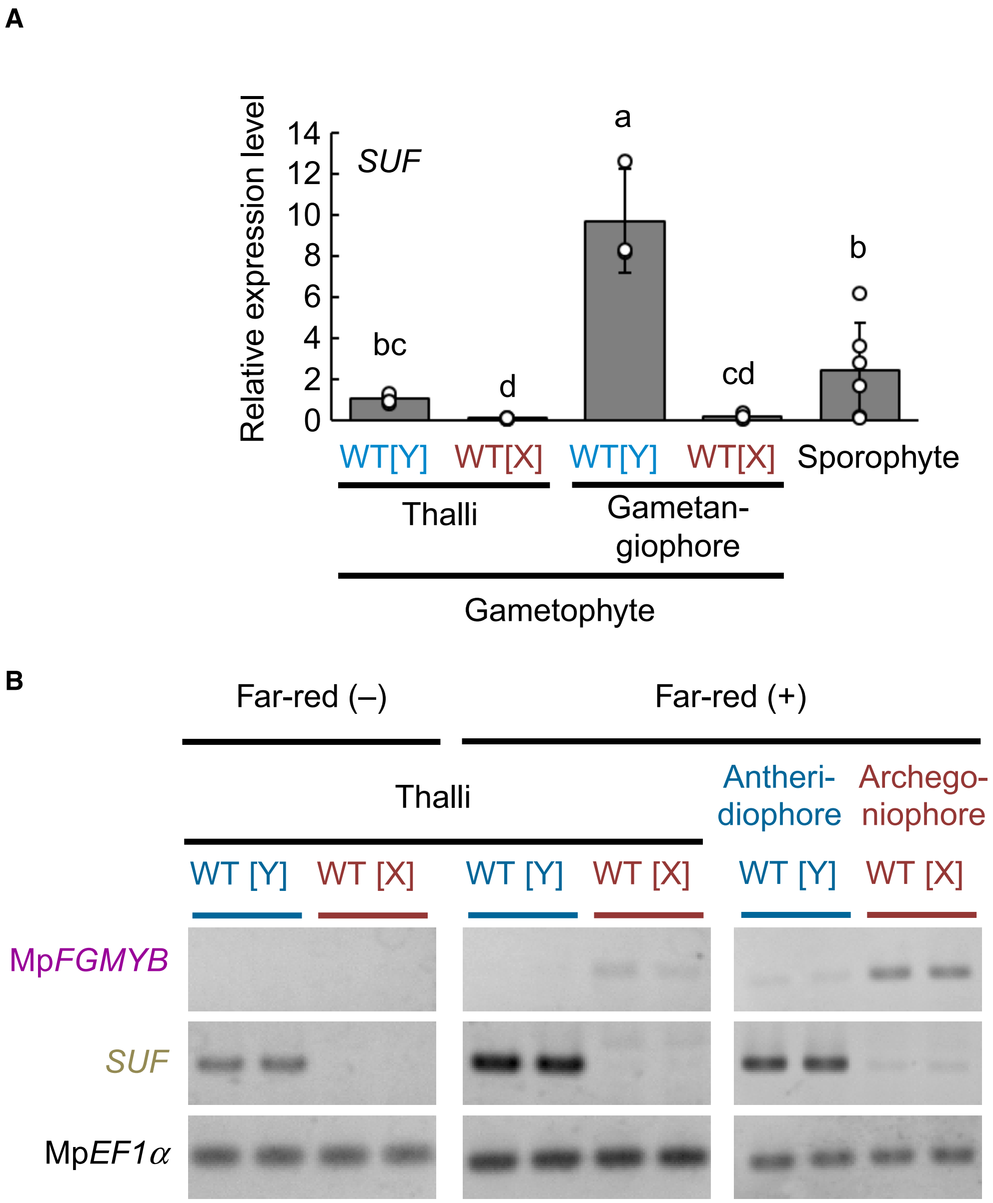

Figure EV4. Expression analyses of *SUF*.

A Real-time RT–PCR analyses indicating preferential accumulation of *SUF* transcripts in male reproductive organs. Constitutively expressed Mp*EF1α* was used for normalization. Measurements of six biological replicates for thalli and sporophyte, and three biological replicates for gametangiophores are plotted. Bars represent mean ± SD. Symbols above bars indicate grouping by $P < 0.05$ in a Tukey–Kramer test. See Source Data online for measurements and statistics.

B Strand-specific RT–PCR confirmed male-specific accumulation of *SUF* transcripts in vegetative and reproductive organs, regardless of the induction of reproductive growth by far-red irradiation. Biological duplicates were analyzed for each sex. Mp*EF1α* was used as a control.

Source data are available online for this figure.

 © 2018 The Authors

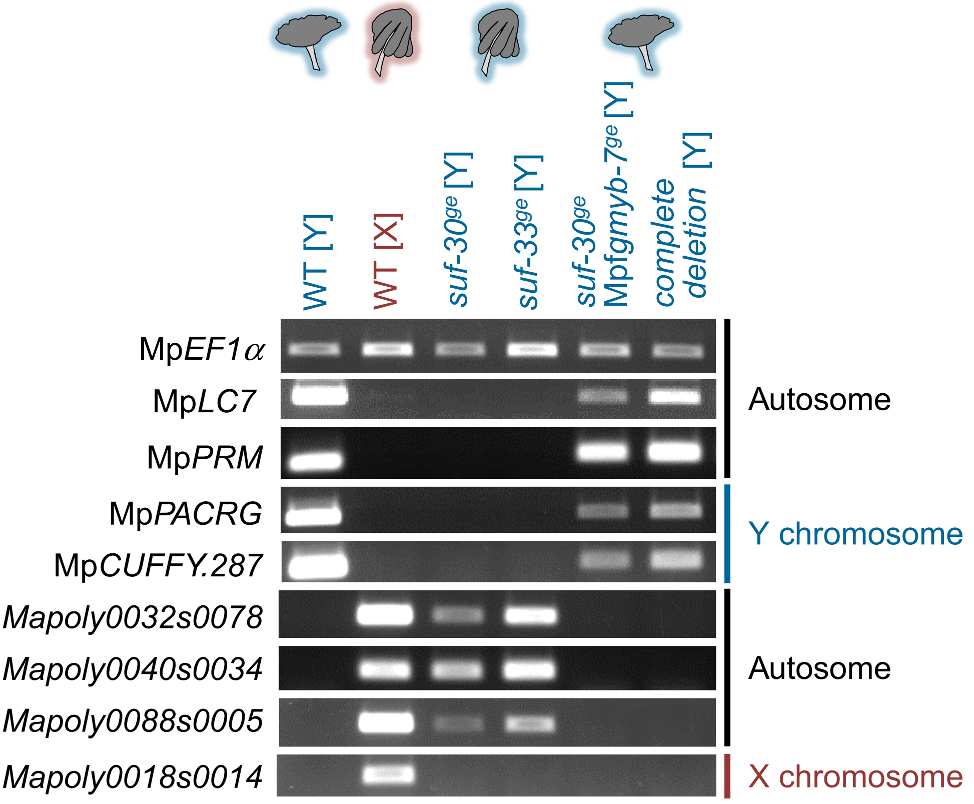

Figure EV5. Expression analyses of sex-specific genes in *suf* [Y].

RT–PCR analyses showing feminized gene expression patterns of *suf* [Y]. Note that the expression of female-specific genes in *suf* [Y] was suppressed by an additional mutation in the Mp*FGMYB*-coding region. Mp*EF1α* was used as a control.

Source data are available online for this figure.
