# Supplementary figures and images for: A cis‐acting bidirectional transcription switch controls sexual dimorphism in the liverwort
Source: EMBO J. 2019 Jan 4;38(6):e100240. doi: 10.15252/embj.2018100240 (PMC6418429; doi:10.15252/embj.2018100240)

Source data for FigEV4B

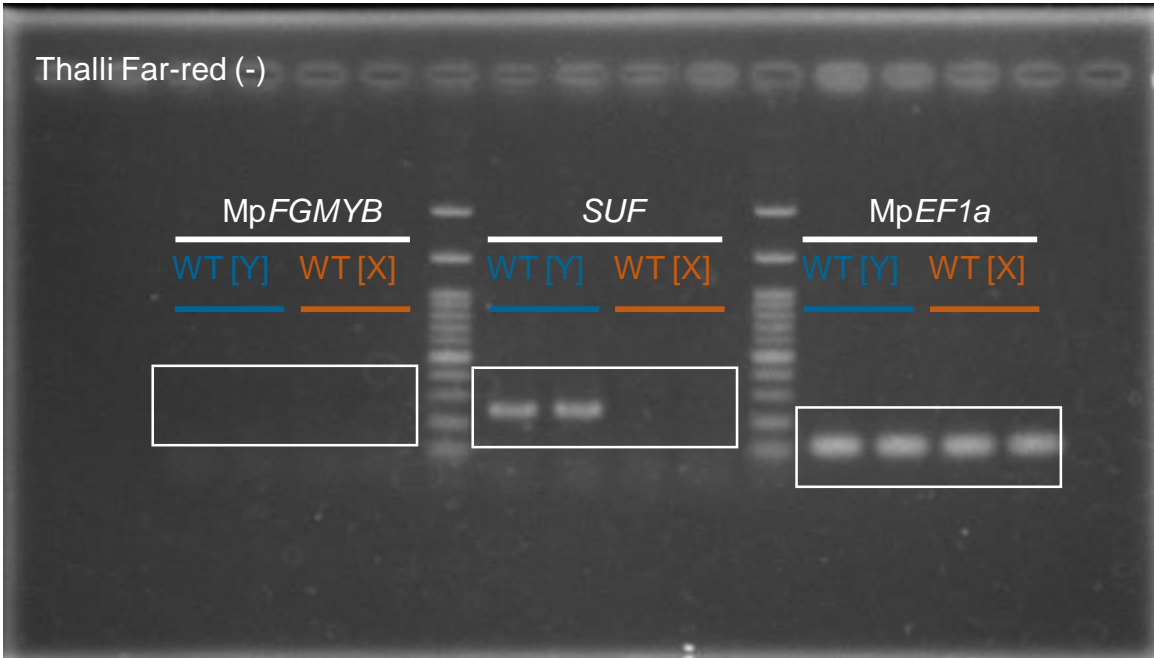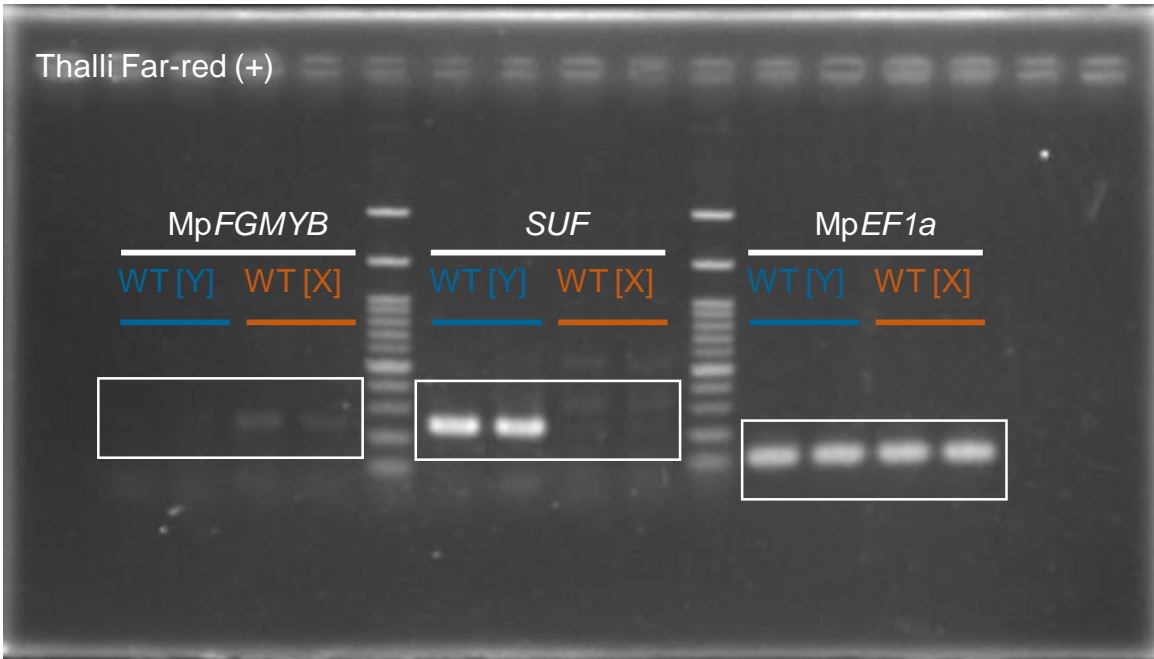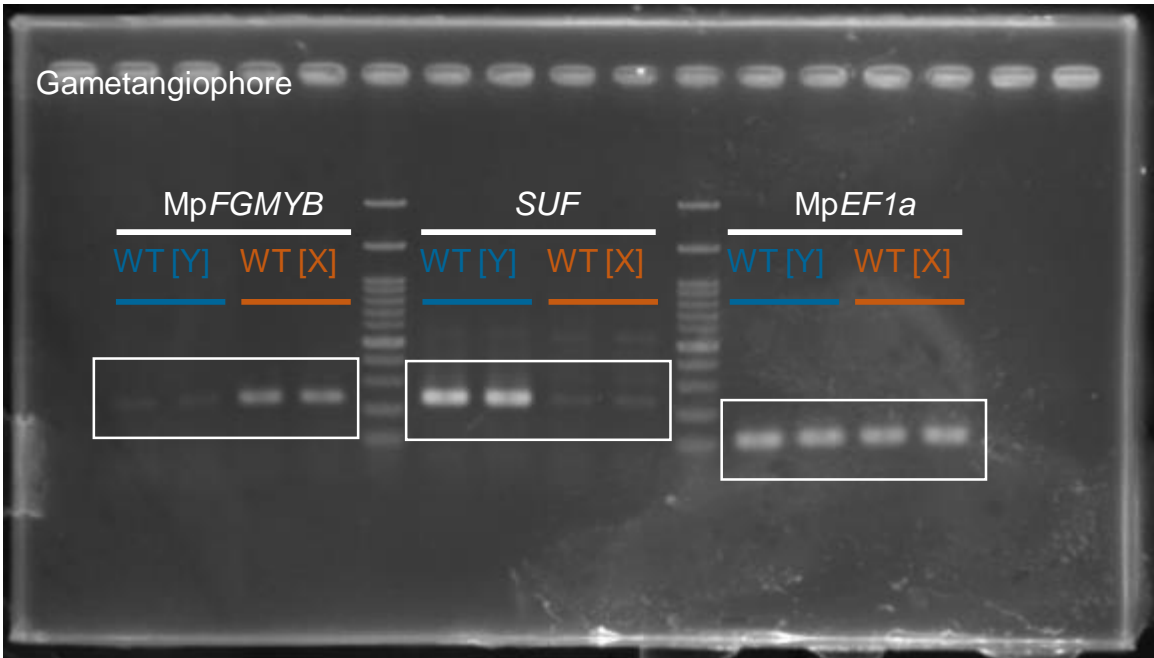

Supplement: Supplementary file 5 — Source Data for Expanded View and Appendix [file EMBJ-38-e100240-s010.zip › EMBOJ-2018-100240R_SourceDataForFigureEV4B.pdf]

Source data for Fig3B Appendix Fig S1C

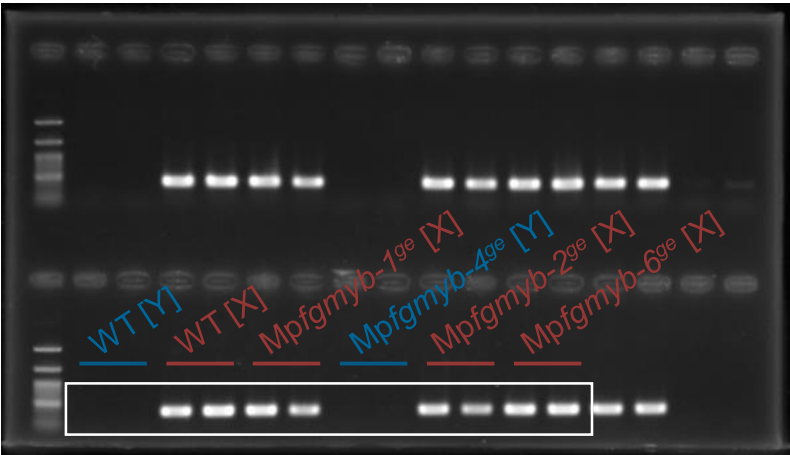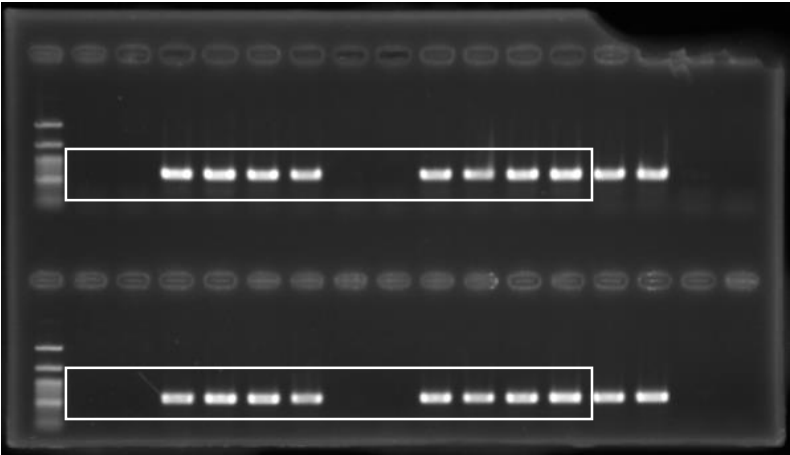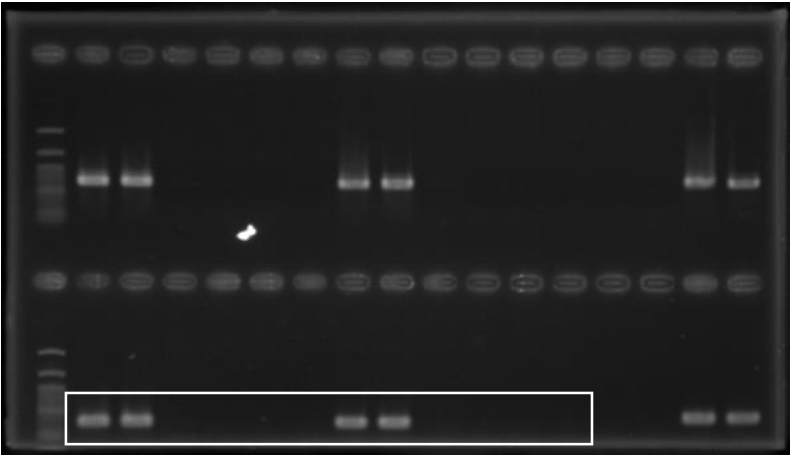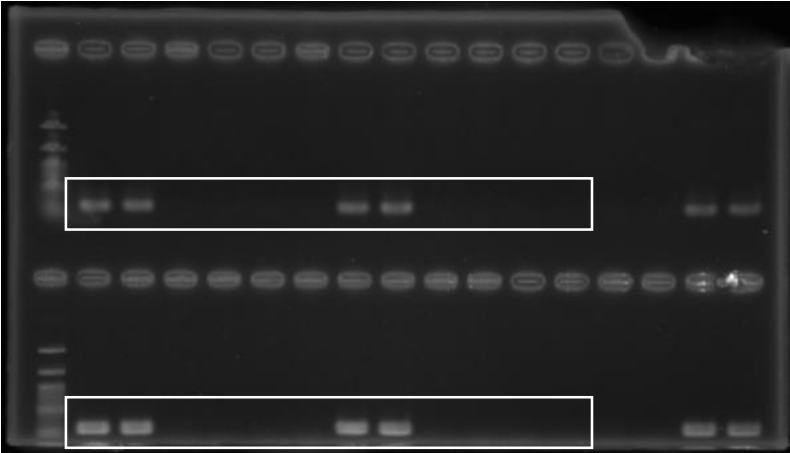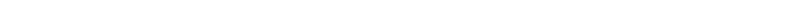

Supplement: Supplementary file 5 — Source Data for Expanded View and Appendix [file EMBJ-38-e100240-s010.zip › EMBOJ-2018-100240R_SourceDataForFigureS1C.pdf]

Fig.EV5

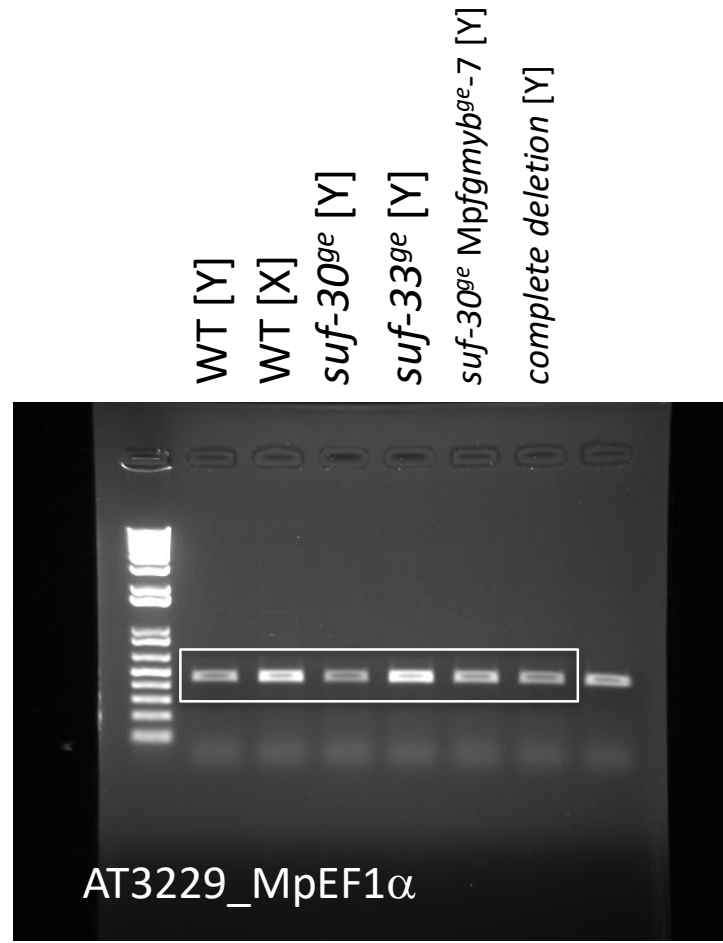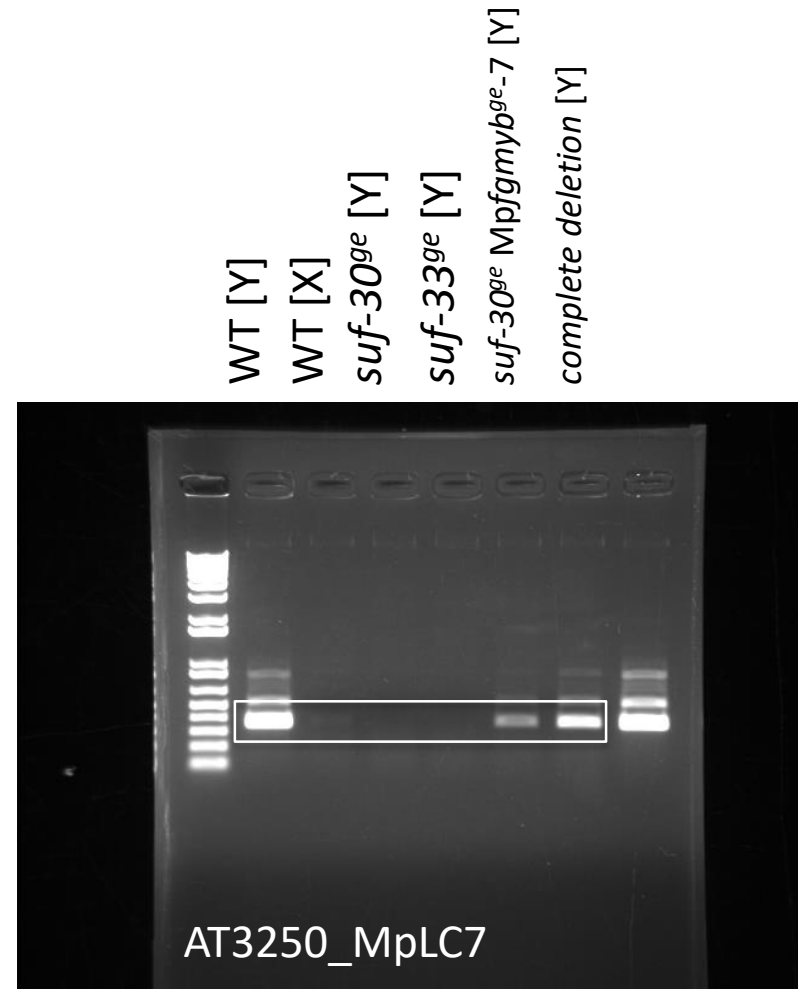

Fig.EV5

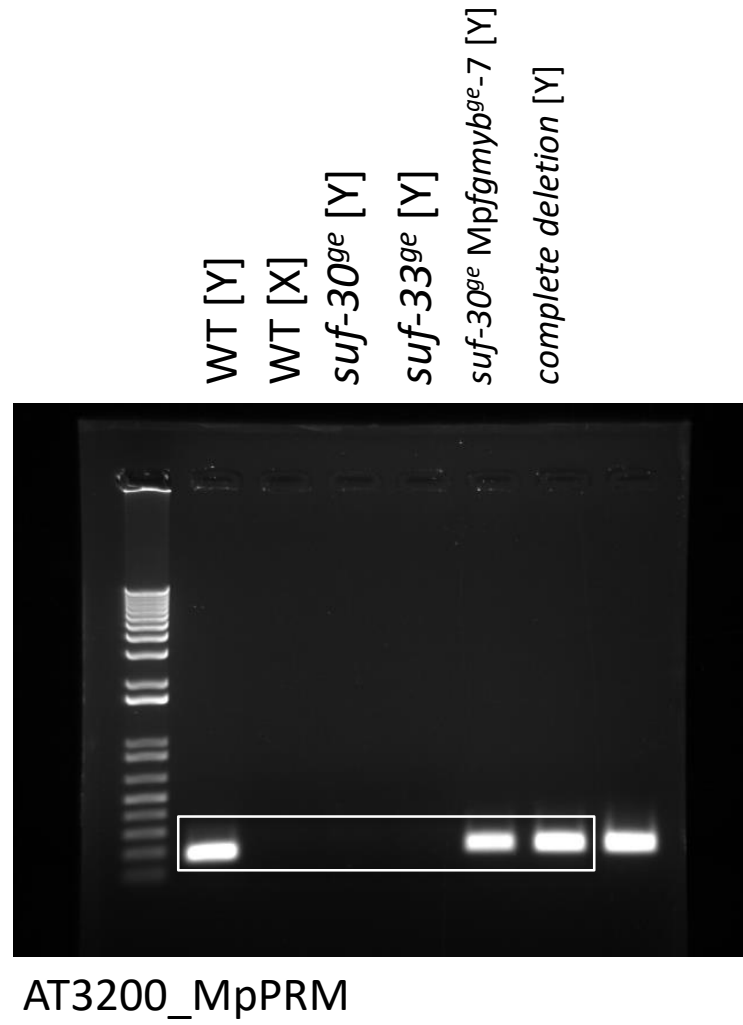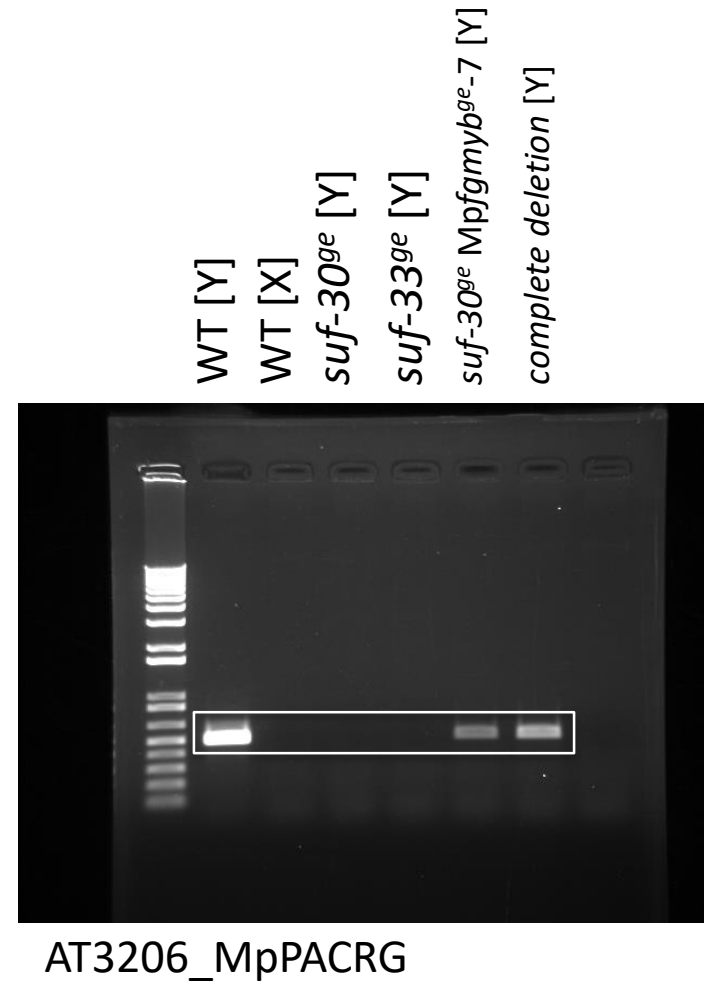

Fig.EV5

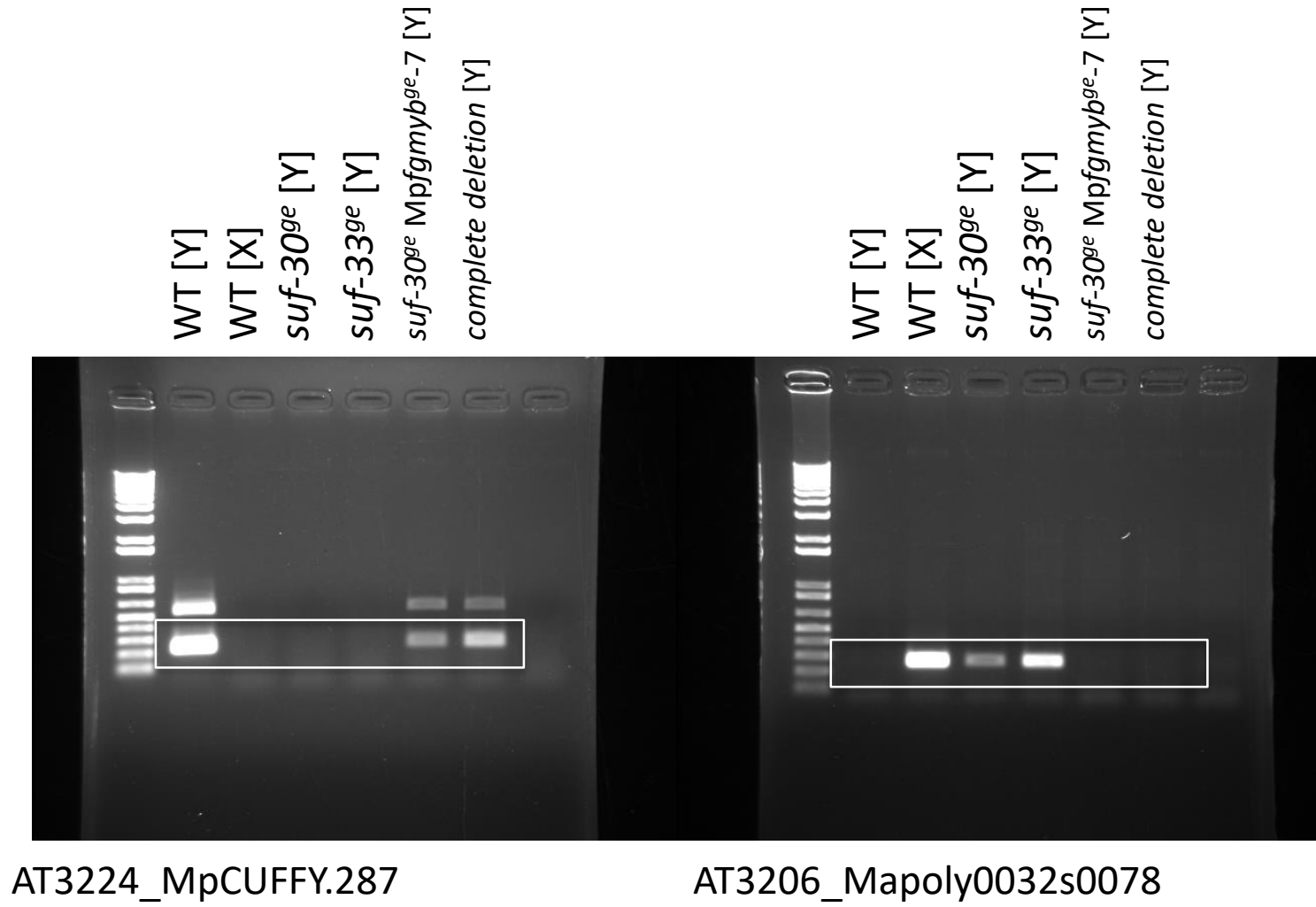

Fig.EV5

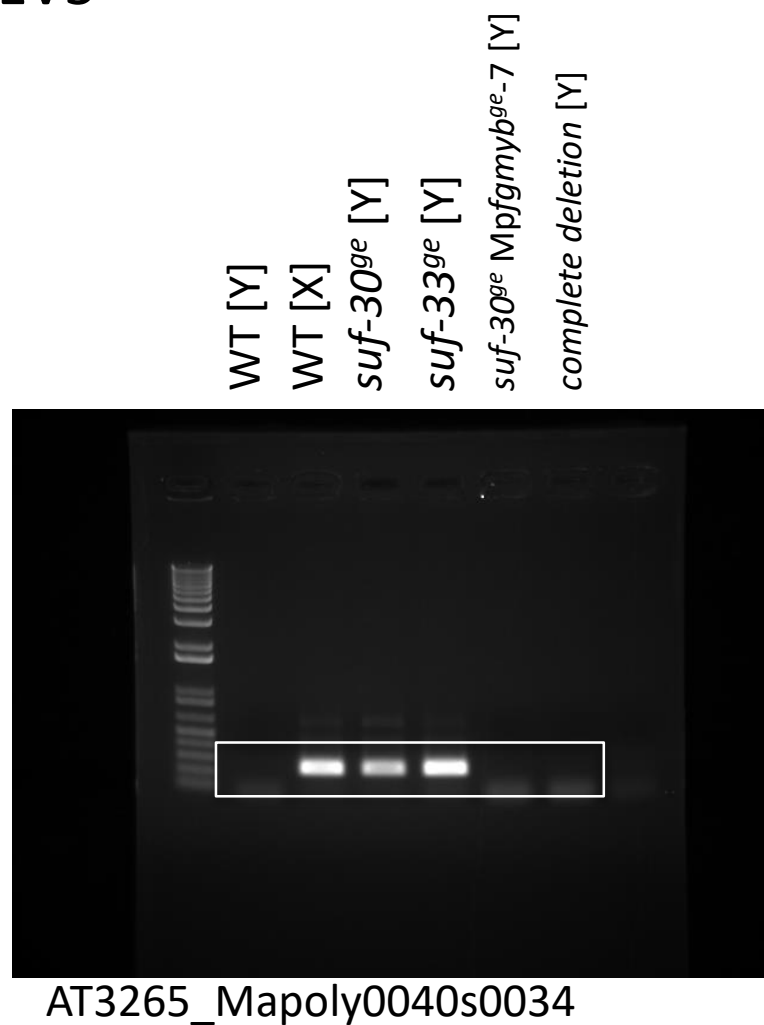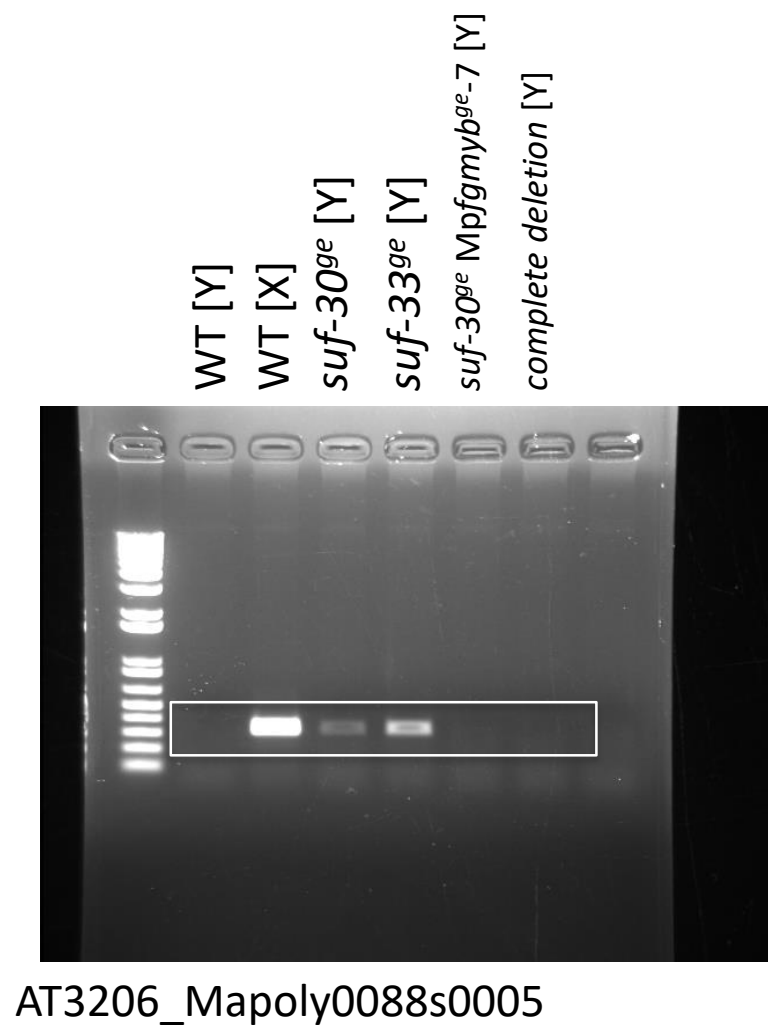

Fig.EV5

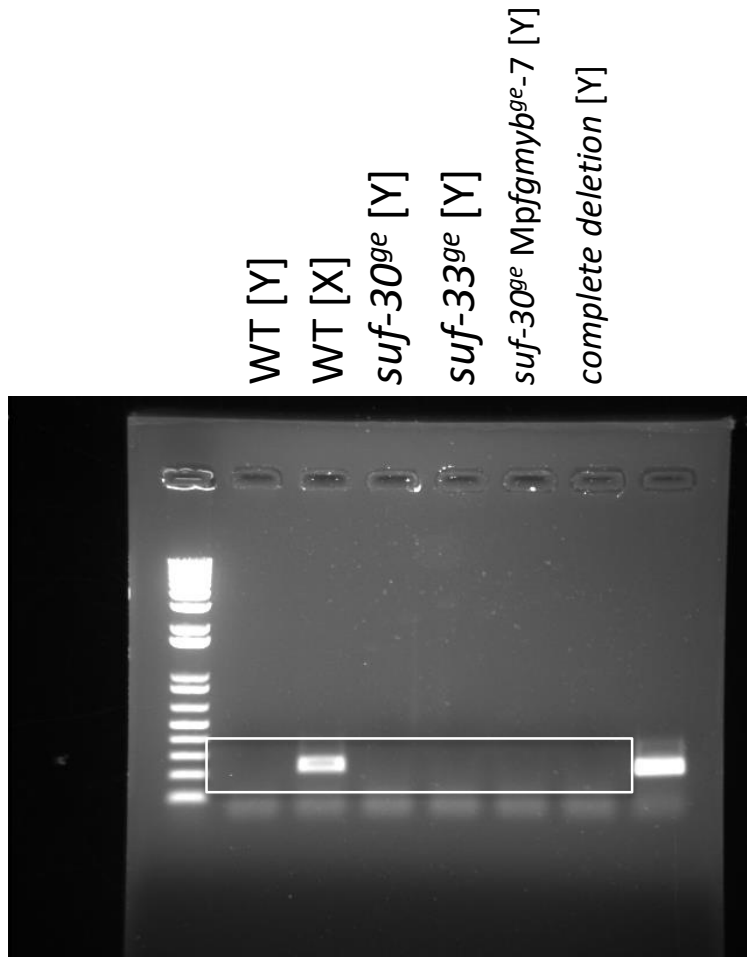

AT3265\_Mapoly0018s0014

Supplement: Supplementary file 5 — Source Data for Expanded View and Appendix [file EMBJ-38-e100240-s010.zip › EMBOJ-2018-100240R_SourceDataForFigureEV5.pdf]

Source data for Fig EV3A

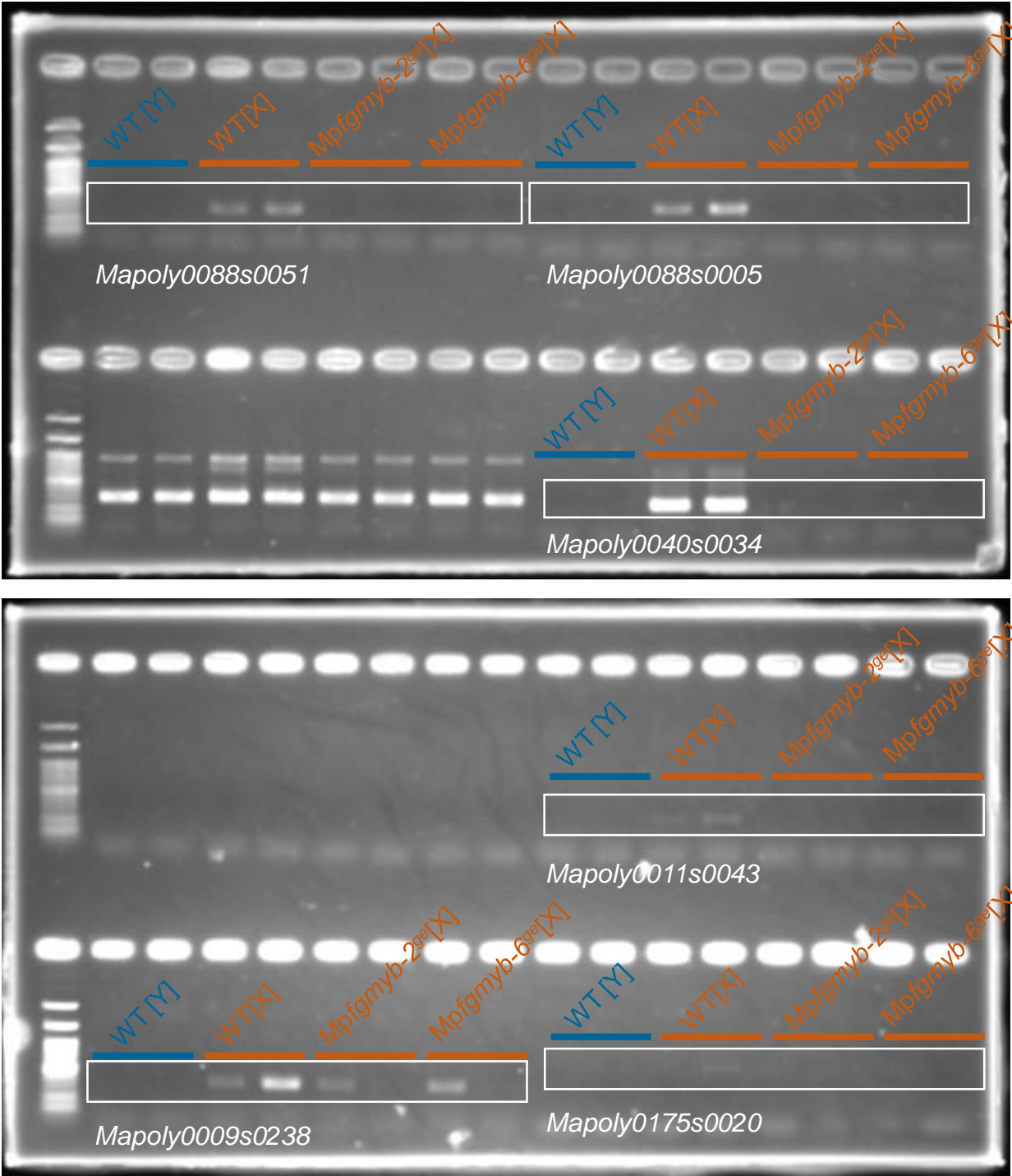

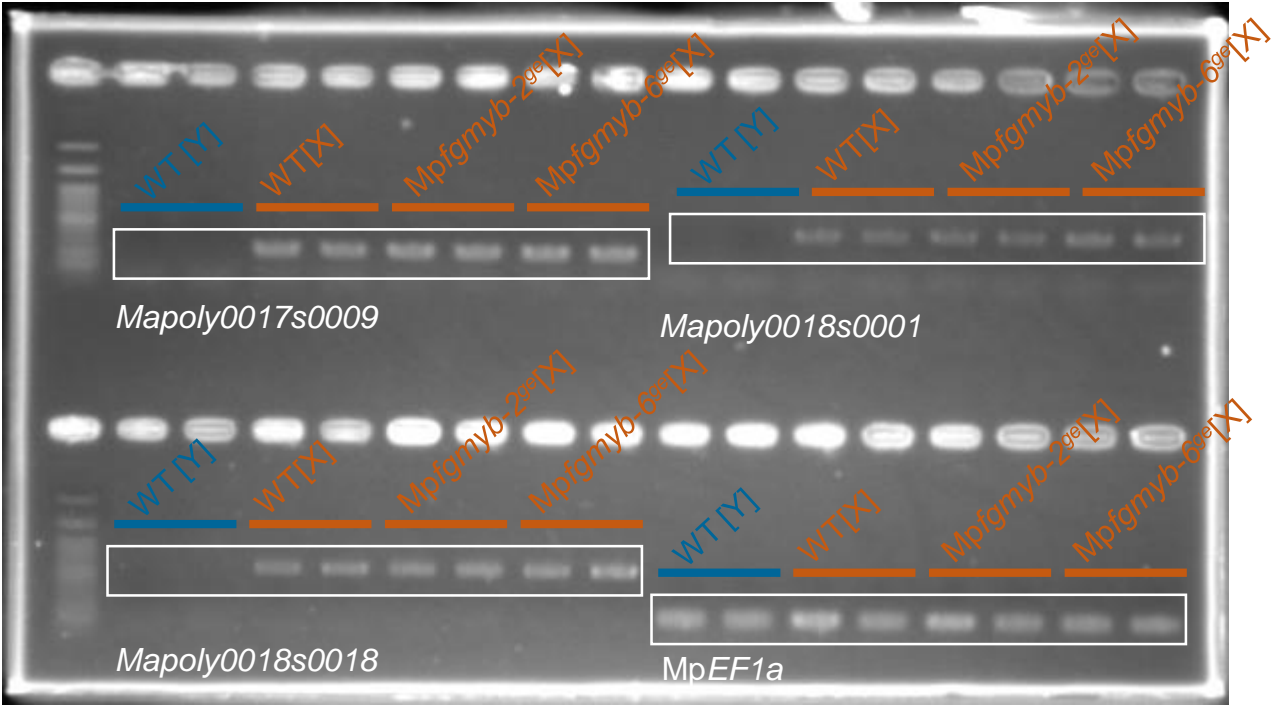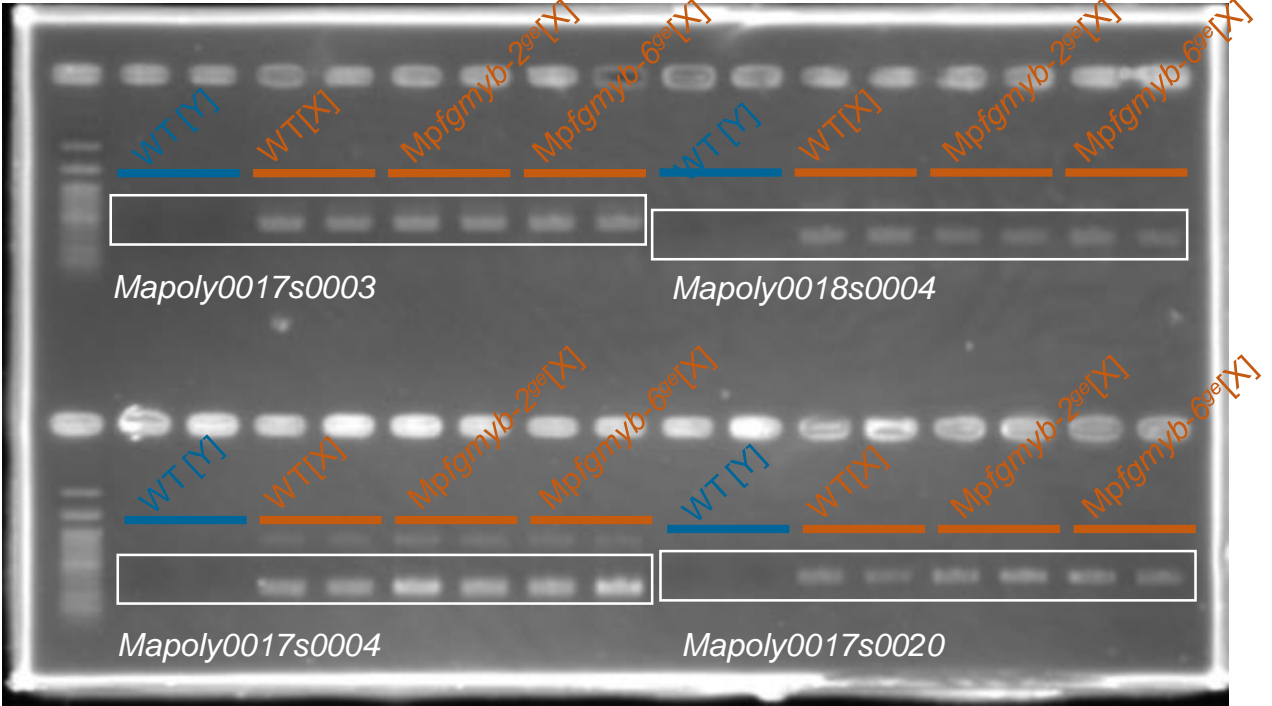

Supplement: Supplementary file 5 — Source Data for Expanded View and Appendix [file EMBJ-38-e100240-s010.zip › EMBOJ-2018-100240R_SourceDataForFigureEV3A.pdf]

Source data for Fig 2C

MpFGMYB

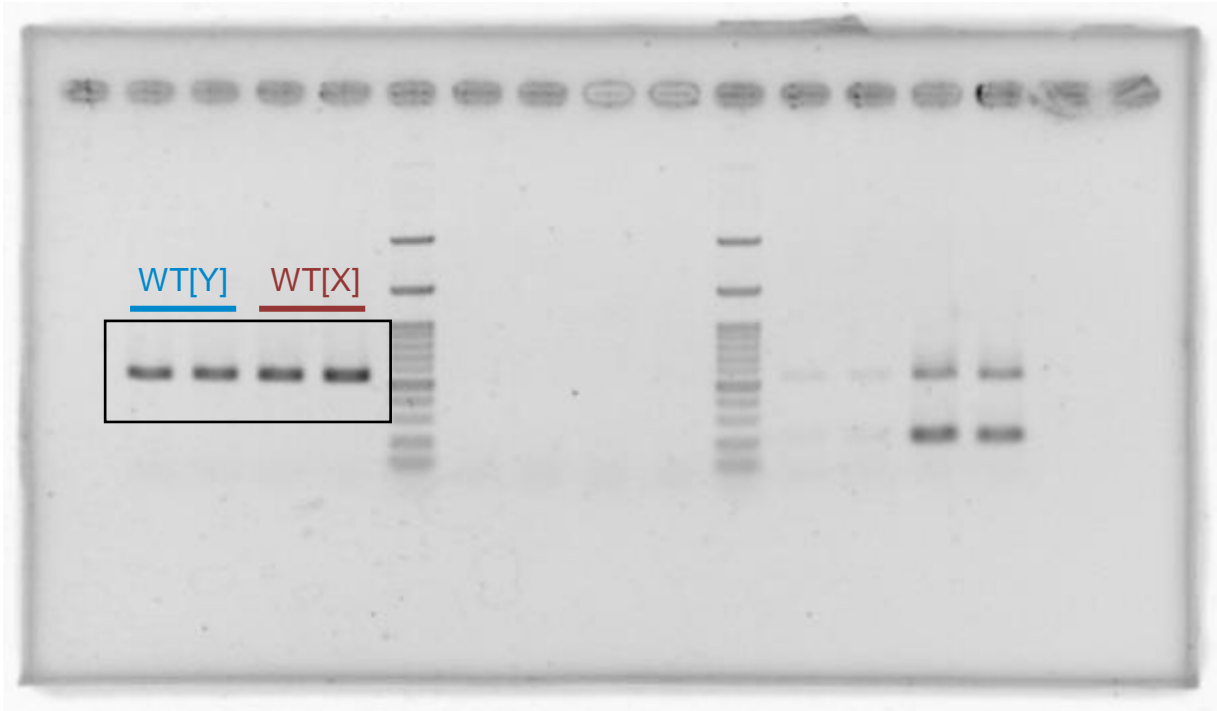

MpEF1a

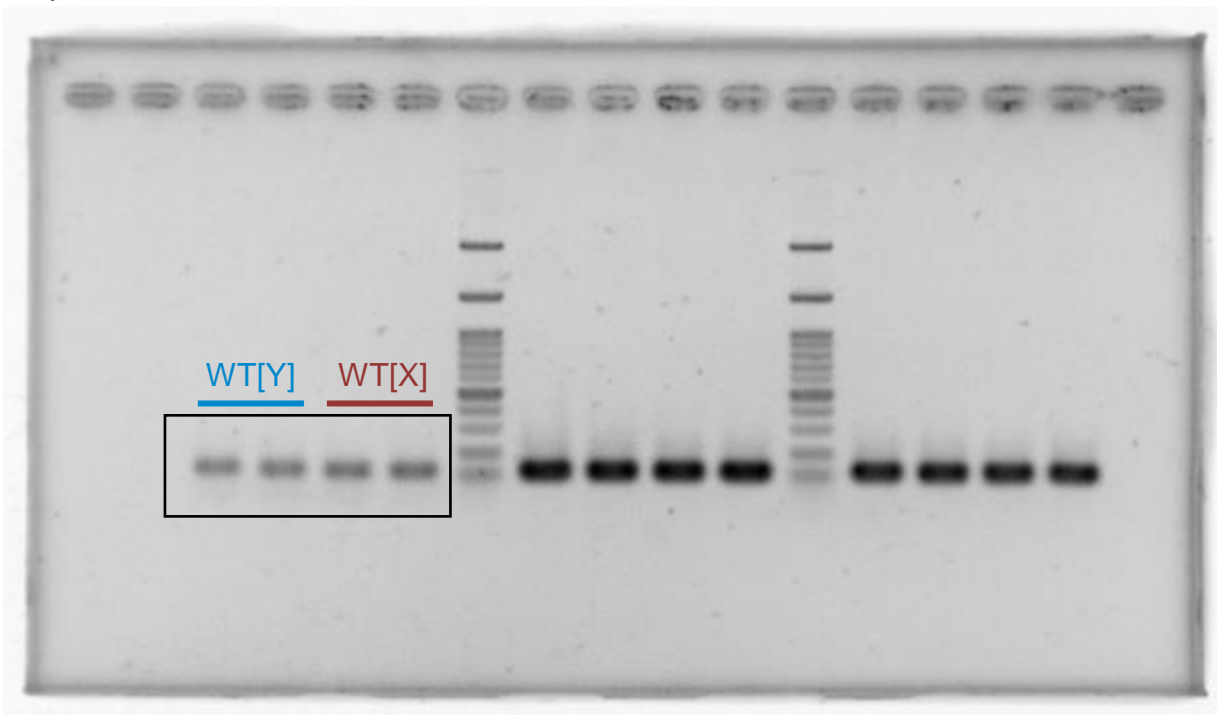

Supplement: Supplementary file 7 — Source Data for Figure 2 [file EMBJ-38-e100240-s005.zip › embj2018100240-sup-0006-SDataFig2C.pdf]

Source data for Fig3B

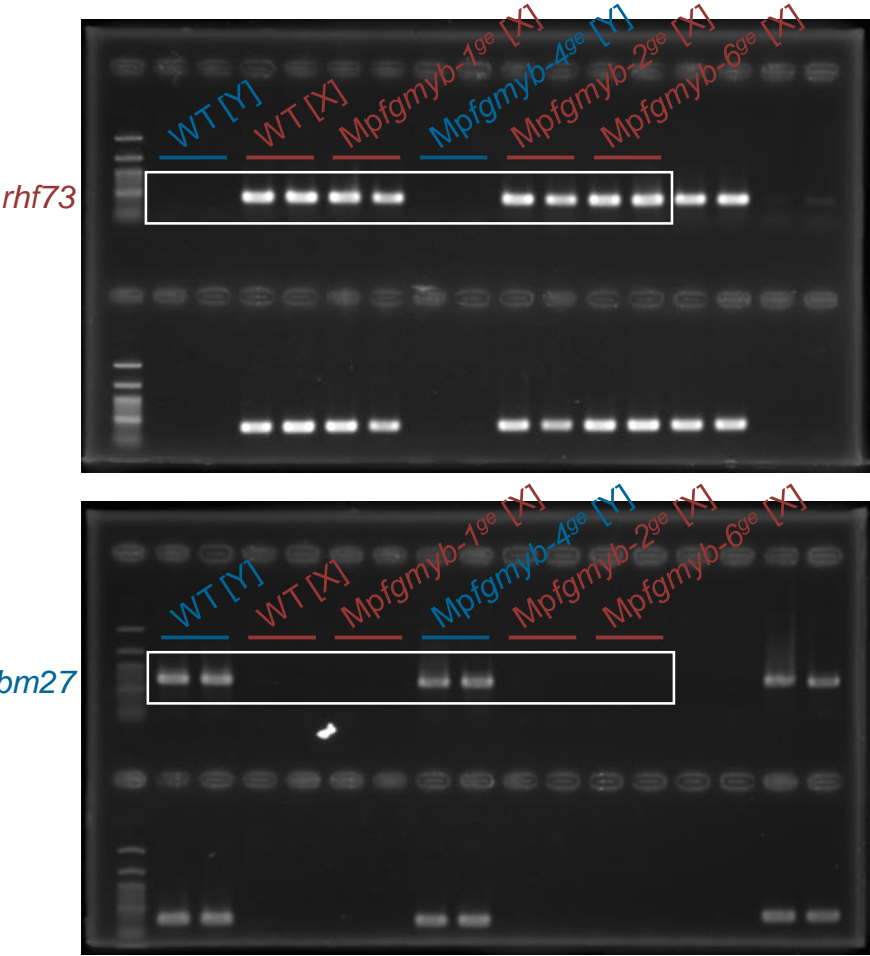

Supplement: Supplementary file 8 — Source Data for Figure 3 [file EMBJ-38-e100240-s006.pdf]

Source data for Fig 4D

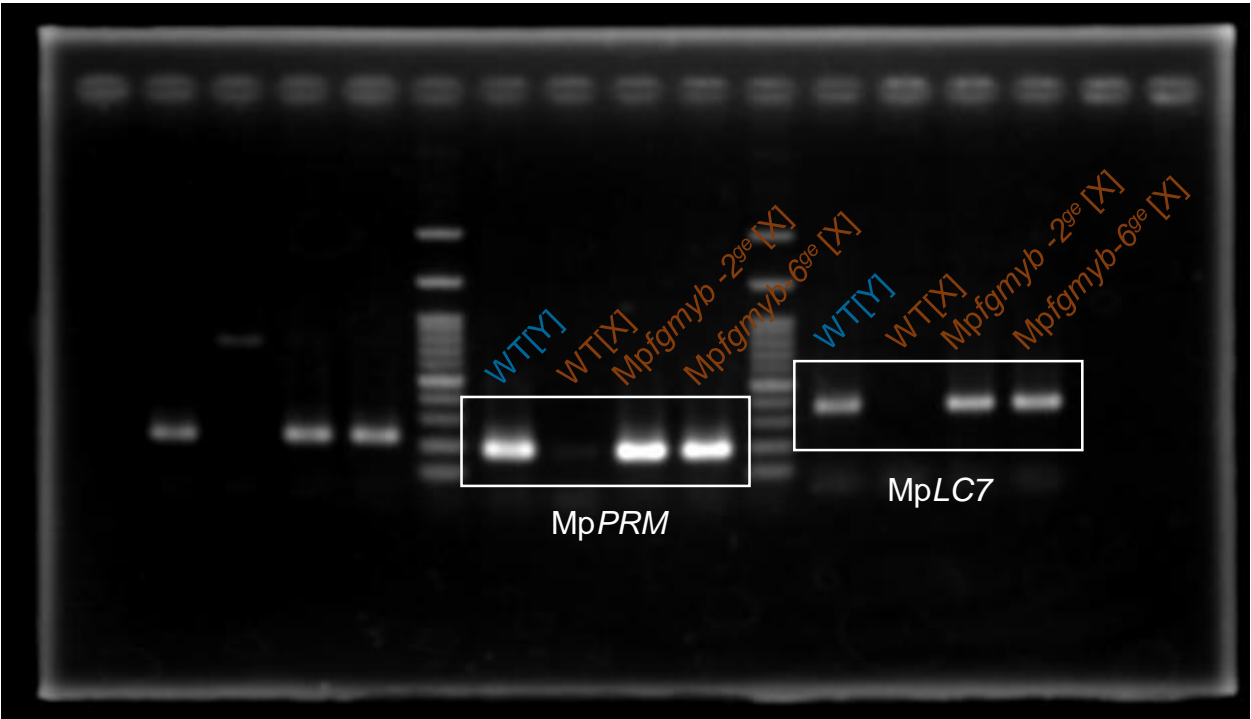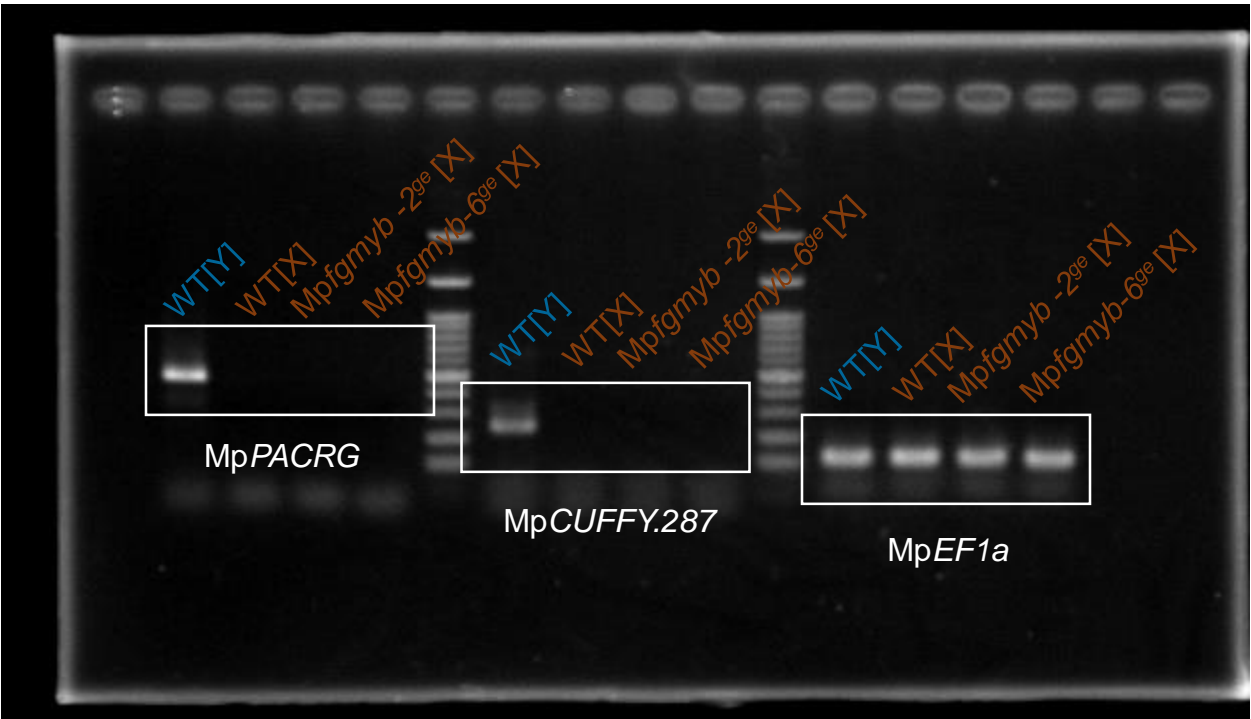

Supplement: Supplementary file 9 — Source Data for Figure 4 [file EMBJ-38-e100240-s007.pdf]

Source data for Fig 5B

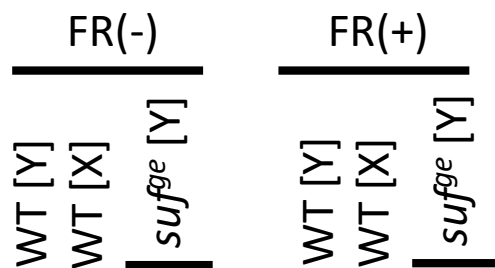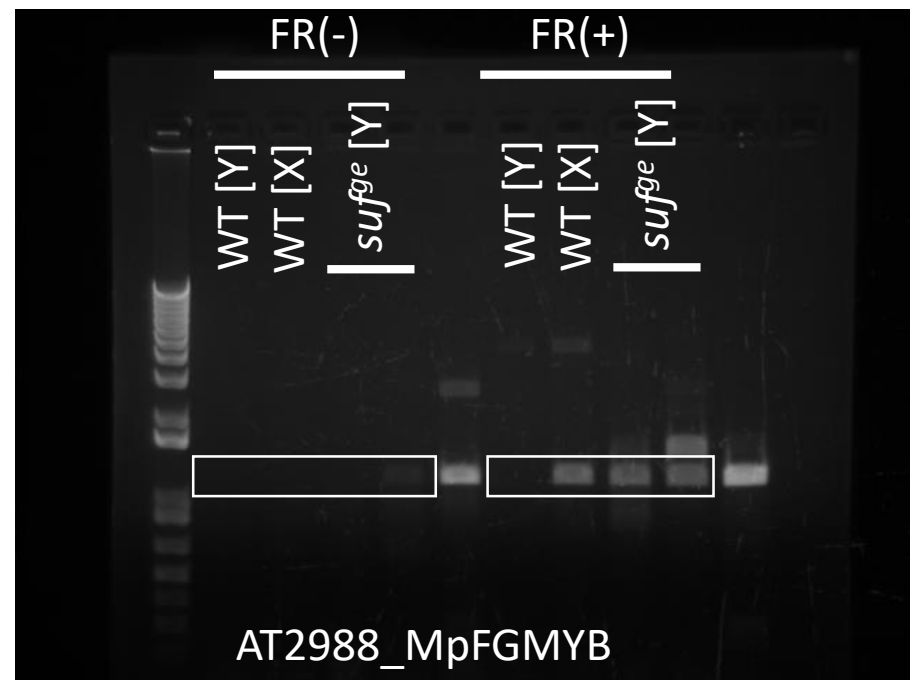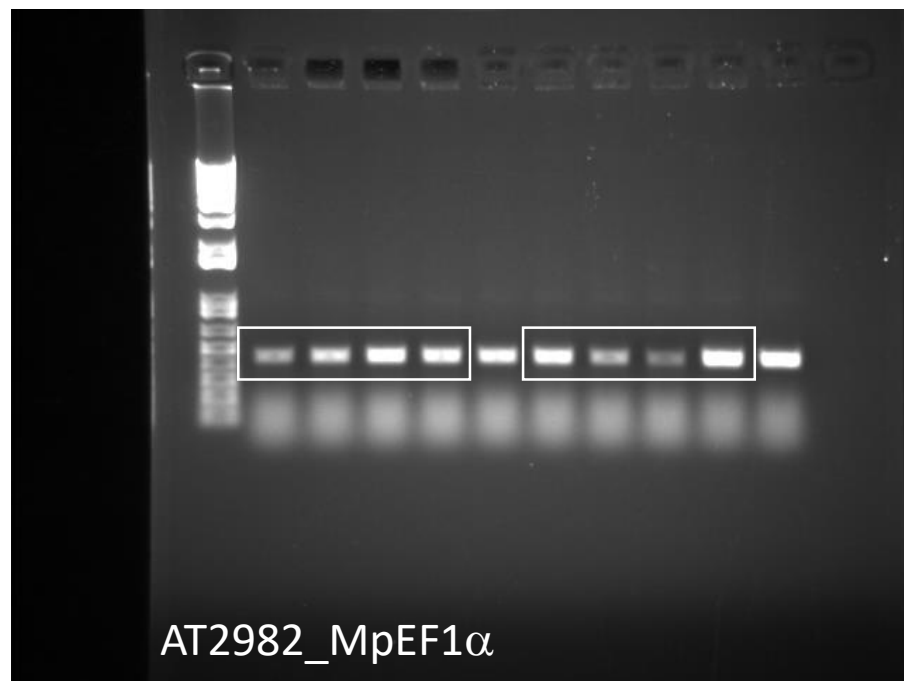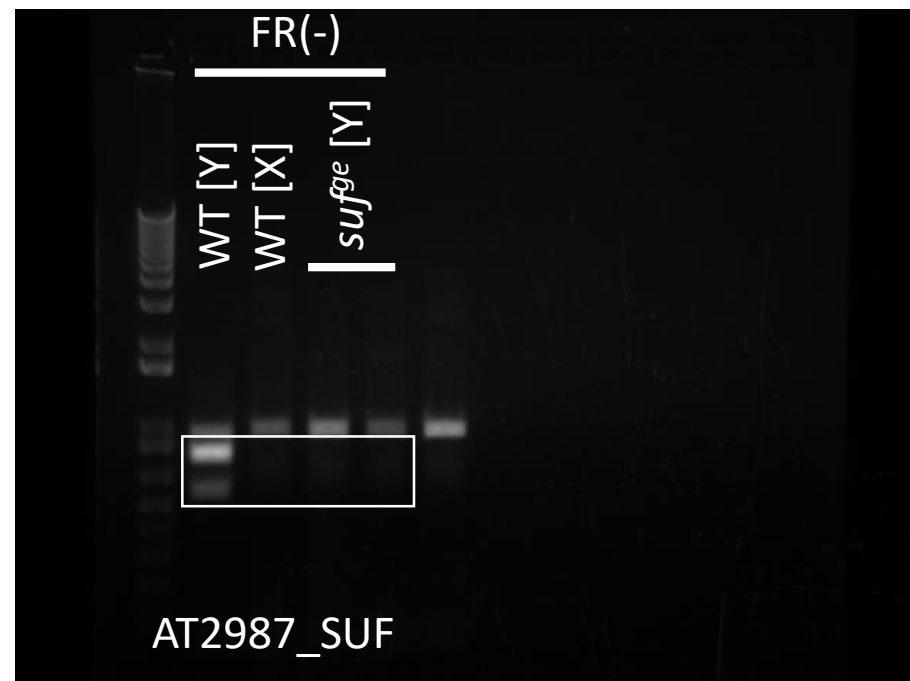

Supplement: Supplementary file 10 — Source Data for Figure 5 [file EMBJ-38-e100240-s008.pdf]
